# Supplementary material for: Artificial intelligence-based analytics for impacts of COVID-19 and online learning on college students’ mental health
Source: PLoS One. 2022 Nov 18;17(11):e0276767. doi: 10.1371/journal.pone.0276767 (PMC9674166; doi:10.1371/journal.pone.0276767)
Supplement: S1 Data — (ZIP) [file pone.0276767.s001.zip › Revised/Supporting Information.pdf]

Artificial Intelligence-Based Analytics for Impacts of COVID-19 and Online Learning on College Students' Mental Health

Mostafa Rezapour[1,*,†], Scott K. Elmshaeuser[1]

1 Department of Mathematics, Wake Forest University, Winston-Salem, NC, U.S.
* rezapom@wfu.edu

†The corresponding author conducted this research while he was working in the department of mathematics. His current affiliation: Biomedical Informatics, Wake Forest University School of Medicine, Winston-Salem, NC, U.S. (mrezapou@wakehealth.edu).

Supporting Information

Data Preprocessing.

In this subsection, we describe how the US-FD1W-25(d) data set is derived from the original data set Final Dataset 1st Wave (FD1W). To address missing entries in the original dataset, and its subdatasets, we use two primary methods: the deletion of rows with missing values and imputation.

The deletion of rows with missing values from the FD1W and computational results

If we remove all rows of the FD1W that contain at least one missing value, the result is the *Cleaned-FD1W*; however, the *Cleaned-FD1W* is a 55 by 161 tabular dataset, which means that we must deal with a "large-p, small-n" problem (see [**?**]), because the number of predictors (features or columns) is much larger than the number of samples (datapoints or rows).

We first select Question 25a as the target variable, split the data into training and test sets (75%-25%), and train possible supervised multiclass classifiers on the data. We aim to find an accurate model and analyze its feature importance scores. In large-p, small-n cases, one of the most widely used machine learning methods is Support Vector Machine (SVM). Since our target variable, Question 25a, contains five classes, we apply SVM OVO, which is appropriate for multi-class classifications. Due to a severe large-p, small-n problem, the SVM OVO model obtains a test accuracy score of 52.63%. Since the accuracy of the SVM OVO model is not satisfactory, we do not analyze the feature importance scores of the model. We also apply the XGBoost model with k-fold cross validation, where k changes between 2 and 20. Since there are many more features than datapoints, the obtained accuracy scores for the model are not satisfactory. Figure 1 displays accuracy scores of XGBoost using k-fold cross validation for multiple numbers of folds.

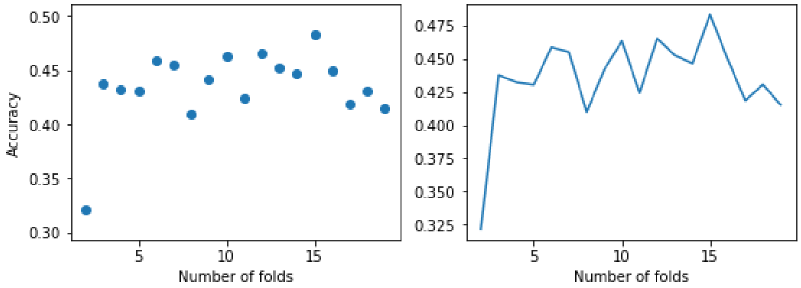

Fig 1. Accuracy scores of XGBoost with k-fold cross validation on the *Cleaned-FD1W*.

We then use CatBoost with multiple numbers of trees. Figure 2 displays accuracy scores of CatBoost with multiple numbers of trees. It turns out that the CatBoost with 19 trees outperforms with an accuracy score of 47.2%; however, due to large-p, small-n related issues, the accuracy for boosting models on the *Cleaned-FD1W* is not satisfactory either. To alleviate large-p, small-n related issues, where p=160 and n=55, we can reduce the feature dimension by removing unrelated features (columns) from the dataset. So we must determine which features are the least-related features for XGBoost using k=15 folds and CatBoost with 19 trees. Figures 3 and 4 display feature importance scores for XGBoost and CatBoost, respectively.

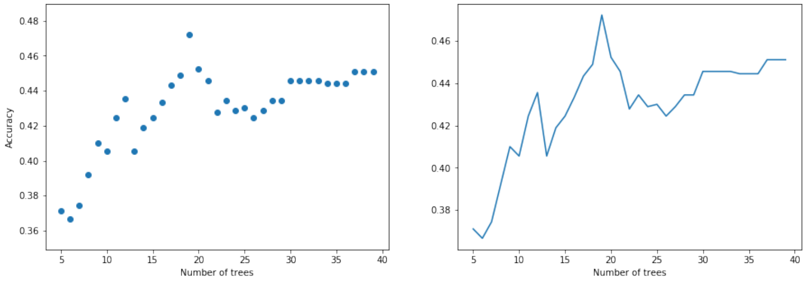

Fig 2. Hyperparameter tuning: Accuracy scores of CatBoost with multiple numbers of trees on the *Cleaned-FD1W*.

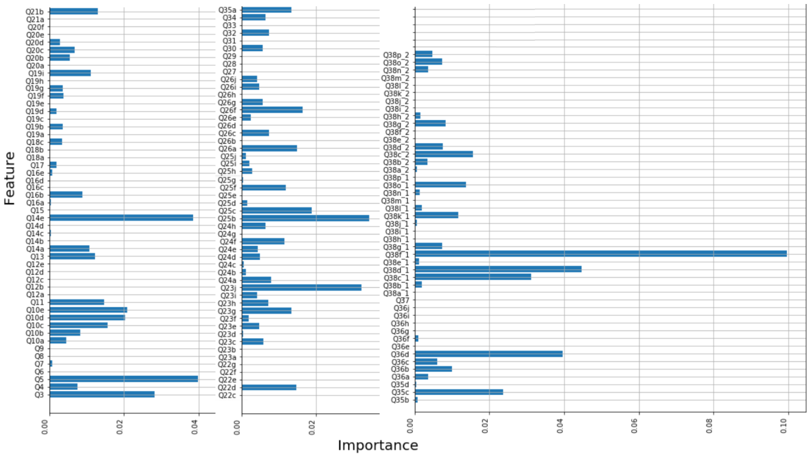

Fig 3. Feature importance scores for XGBoost using k=15 cross-validation on the *Cleaned-FD1W*.

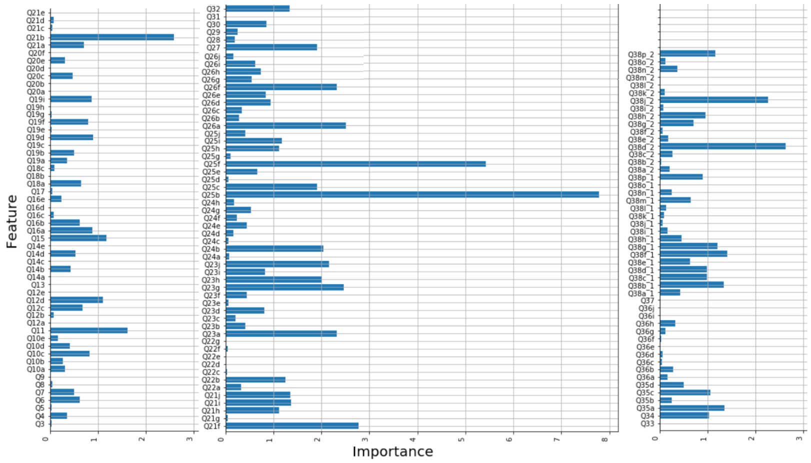

Fig 4. Feature importance scores for CatBoost with 19 trees on the *Cleaned-FD1W*.

Using Figures 3 and 4, we retain the most important features and remove unrelated features, then retrain a CatBoost with 19 trees to calculate feature importance scores (see Figure 5).

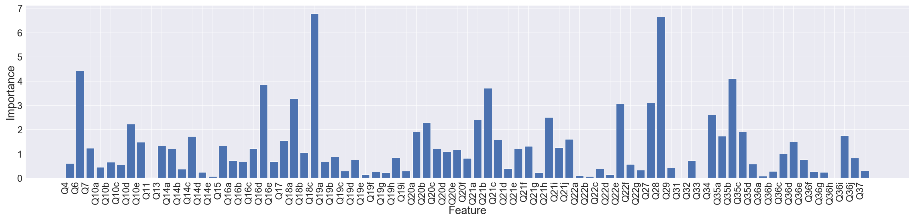

Fig 5. Feature importance scores for CatBoost with 19 trees on the *Cleaned-FD1W* using the related features.

Figure 5 indicates that the dataset still contains some features that can be removed

to reduce the large-p, small-n issues. Based on the feature importance scores in Figure 5, we remove features that do not significantly contribute to the model accuracy. We then retrain the CatBoost with 19 trees and obtain the feature importance scores displayed in Figure 6.

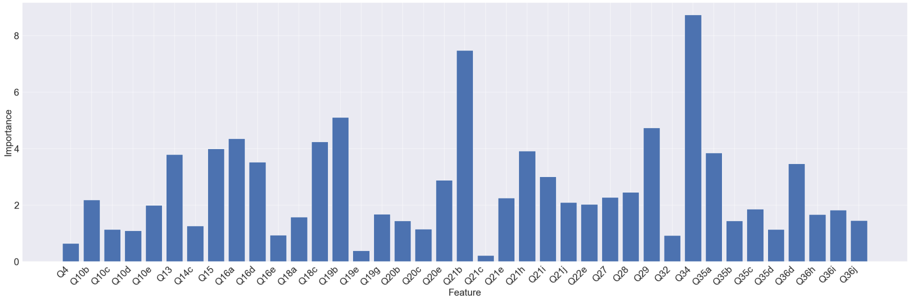

Fig 6. Feature importance scores for CatBoost with 19 trees on the *Cleaned-FD1W* using the most related features.

For a better understanding of the relationships among these variables in the original dataset, the *FD1W*, a correlation heat map is provided in Figure 7.

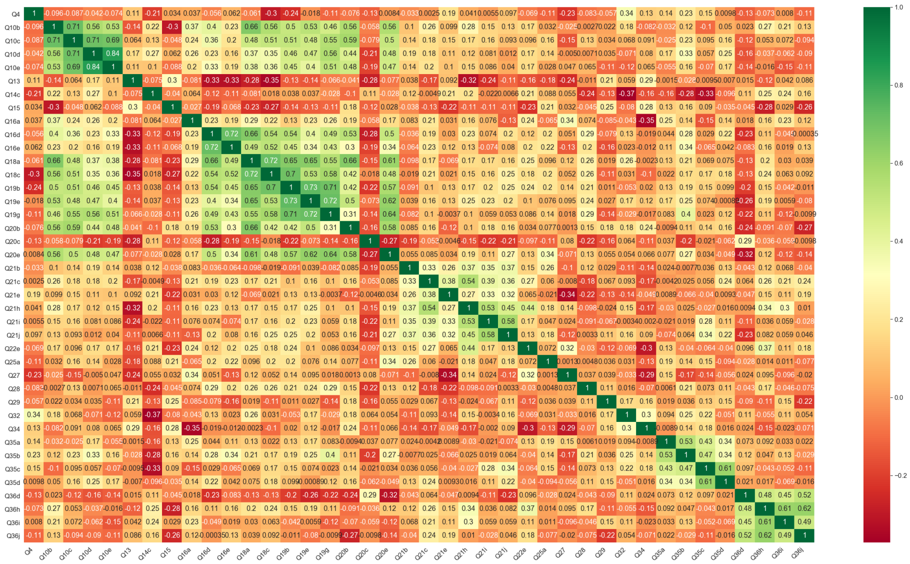

Fig 7. The Correlation Heat map for the relationships between some variables in the original dataset (*FD1W*).

Figures 6 and 7 indicate that the features most related to Question 25a are Questions 4, 13, 34, 21-b, 29, 19-b, 16-a, 18-a, 35-a, 35-d, 20-e and 36-d, and 36-j (see Figure 7).

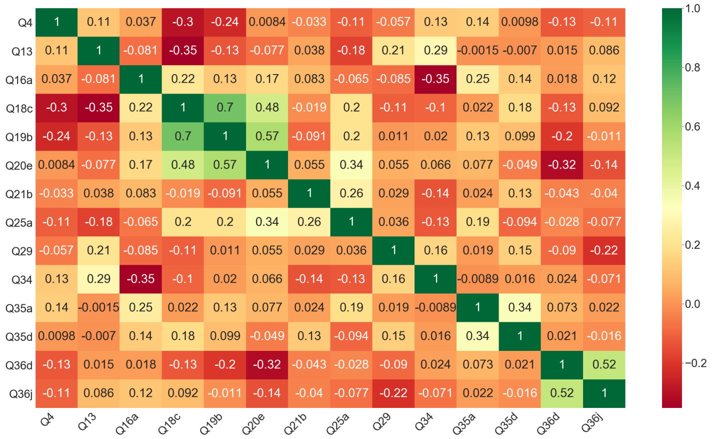

Fig 8. The Correlation Heat map for the relationships between the features most related to Question 25a in the original dataset (*FD1W*).

Since the target variable, Question 25, and all variables in Figure 8 are categorical (nominal or ordinal), we apply a chi-squared test under the null hypothesis H_0^i : Question 25a is independent of Question i, where $i \in X = \{4, 13, 16\text{-a}, 18\text{-c}, 19\text{-b}, 20\text{-e}, 21\text{-b}, 29, 34, 35\text{-a}, 35\text{-d}, 36\text{-d}, 36\text{-j}\}$ (some important categorical features) on the original dataset *FD1W*. By a significance level of $\alpha = 0.05$, the null hypothesis H_0^i is rejected for all $i \in X$ with a p-value less than 0.01; however, none of supervised multiclass classifiers could obtain a satisfactory accuracy on the *Cleaned-FD1W*, but we determined the features most related to our target variables: Question 25a and Question 25d.

We now retain all important features displayed in Figure 7 as well as Question 25a through Question 25f, and we then remove the remaining features from the original dataset (*FD1W*). We call the resulting subdataset "*Q25a-FD1W*."

The deletion of rows with missing values from *Q25a-FD1W* and computational results

First, we remove all rows containing at least one missing value from *Q25a-FD1W*, resulting in a clean 184 by 51 dataset, which includes responses from Afghanistan, Argentina, Bangladesh, Bosnia and Herzegovina, Brazil, Bulgaria, Chile, China, Croatia, Ecuador, Egypt, Georgia, Greece, Guatemala, Hungary, India, Indonesia, Iran, Italy, Japan, Kenya, Kyrgyzstan, Malaysia, Mexico, Mozambique, Nepal, New Zealand, North Macedonia, Oman, Pakistan, Poland, Portugal, Romania, Spain, Thailand, Turkey, United Arab Emirates, United Kingdom, United States of America, and Uzbekistan.

As Figure 9 illustrates, there is a significant difference in the distribution of the cases in classes of Question 25a in the *Q25a-FD1W* dataset, which means that the dataset is biased toward the class "Sometimes," which may cause poor performance of supervised machine learning models. One way to resample datapoints to train a more accurate machine learning model is Synthetic Minority Oversampling Technique (SMOTE) [**?**].

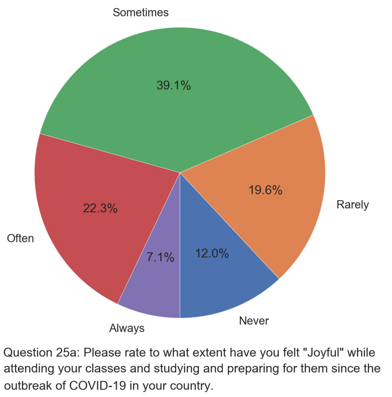

Fig 9. The distribution of examples among classes of Question 25a in the *Q25a-FD1W* dataset.

We first split the data into training-test sets (75%-25%), and then train a Random Forest containing 100 trees (the depth of each tree is 10). Figure 10 displays feature importance scores of the model.

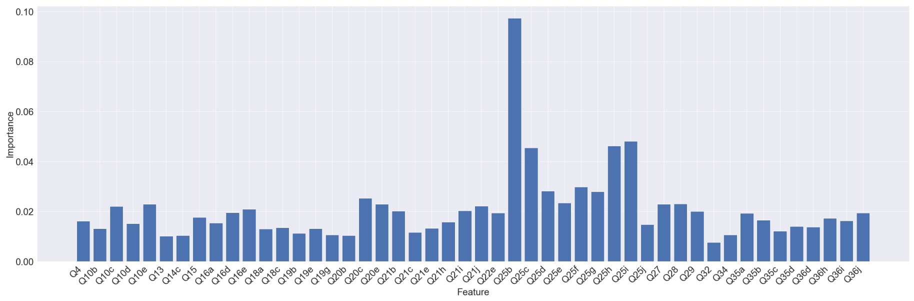

Fig 10. Feature scores of Random Forest with 100 trees and depth equals 10 on the *Q25a-FD1W* dataset.

By deleting of rows with missing values from the *FD1W*, we omit important information. In the following subsection, we change our method for dealing with missing values.

Dealing with missing values of *Q25a-FD1W* by means of KNN imputation method and computational results

In this subsection, we apply KNN imputation method on the "*Q25a-FD1W*" dataset, which contains 237643 missing values. We first fill missing data by means of KNN imputer, split the data into training-test sets (75%-25%), apply SMOTE to the training data set, and finally train a Random Forest with with 200 trees of depth 40. But the model obtains an accuracy of less than 60% on the test set. Figure 11 displays feature importance scores of the model.

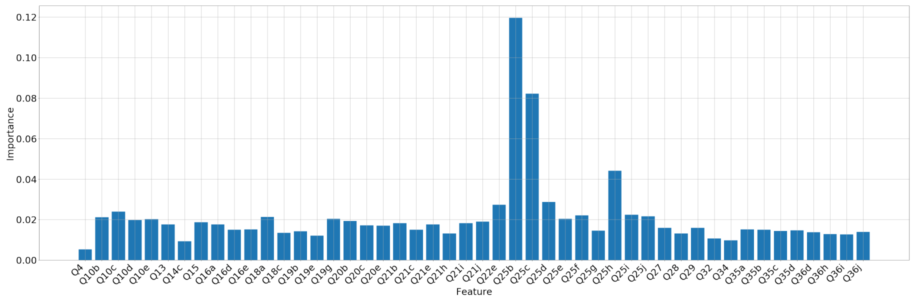

Fig 11. Feature scores of Random Forest with 200 trees and depth equals 40 on the *Q25a-FD1W* dataset after KNN imputation method and SMOTE are applied.

Due to cultural, financial, geographical, and political differences, as well as different approaches to education and different ideas regarding the best philosophy for education in different countries, the utilized supervised machine learning models might not obtain a high accuracy score. To eliminate the aforementioned problem arising from differences among different countries, the next two subsections focus on responses from students in the United States of America.

Machine learning analysis of *US-FD1W-25(a)*

The original dataset *FD1W* contains 392 responses from the US, a 392 by 161 tabular dataset, with 26329 missing values. If we remove all rows with no response to Question 25a, we obtain a 246 by 161 tabular dataset with 6169 missing values; we use the results from previous Subsections, and keep the features most related to Question 25a (and Question 25d), which results in the *US-FD1W-25(a)* dataset. To keep important information and treat missing values appropriately, we use a KNN-imputer, which results in a clean (with no missing values) 246 by 49 tabular dataset; however, the distribution of examples among classes of Question 25a is not even (see Figure 12).

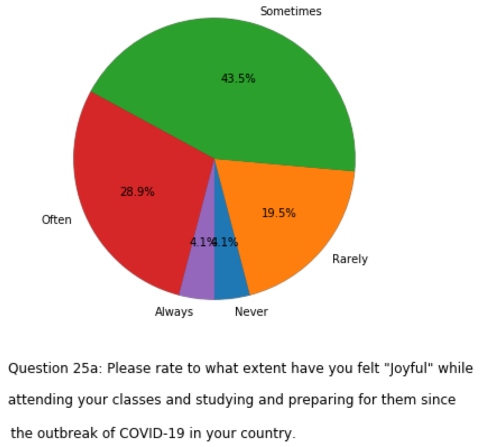

Fig 12. Distribution of examples among Question 25a classes on the *US-FD1W-25(a)* dataset after KNN imputation method is applied.

To train robust models, we split the data into training and test sets (75%-25%), apply SMOTE on the training data, and then train a Random Forest with 200 trees of

depth 40. The Random Forest accuracy score turns out to still be below 65% with feature importance scores displayed in Figure 13.

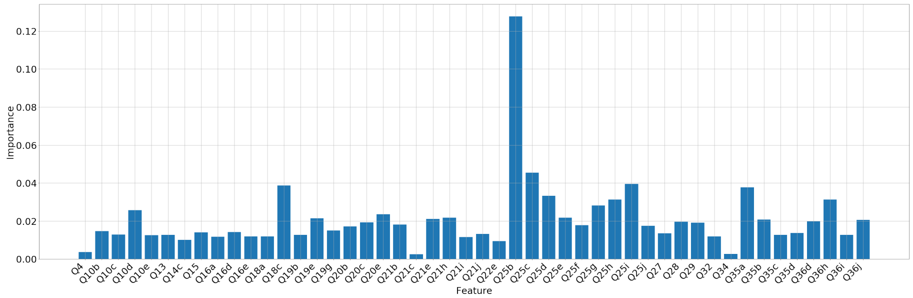

Fig 13. Feature importance scores of the Random Forest with 200 trees of depth 40 on the *US-FD1W-25(a)* dataset after KNN imputation method and SMOTE are applied.

For a better understanding of relationships among some important features, a correlation heat map for some important variables in the *US-FD1W-25(a)* dataset after KNN imputation method is displayed in Figure 14.

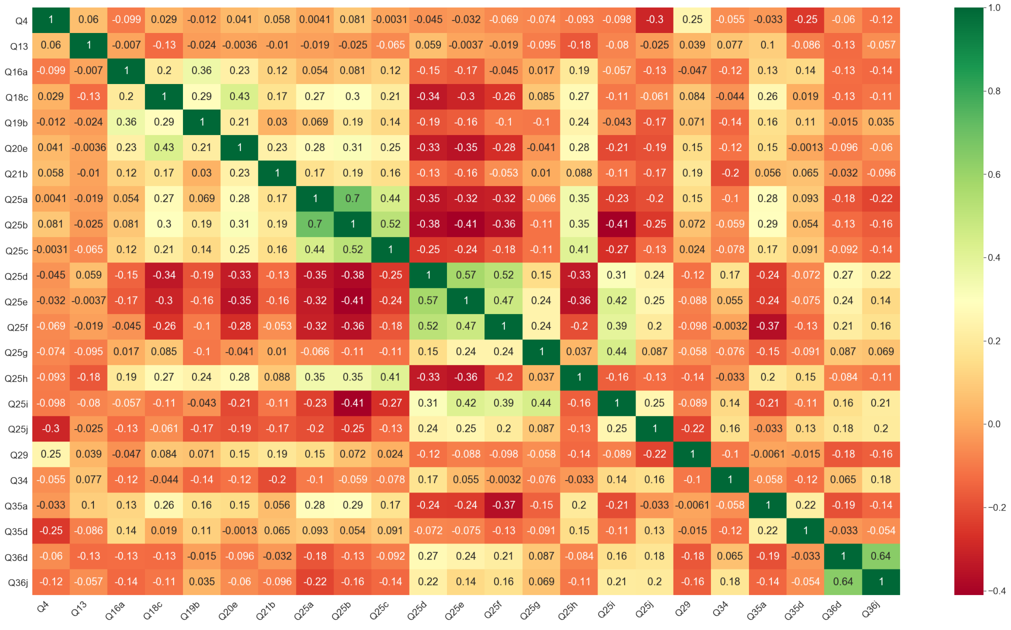

Fig 14. The correlation heat map for some important variables in the *US-FD1W-25(a)* dataset after KNN imputation method.

Question 25d seems to be a better target variable than Question 25a because it correlates more strongly with the other variables. In the next subsection, we switch from examining Question 25a as the target variable to Question 25d because they appear to be opposites. Hence, we consider Question 25d as the target variable and remove all rows with no response for Question 25d from the US-FD1W, and keep all columns corresponding to Questions 4, 10b, 10c, 10d, 10e, 13, 14c, 15, 16a, 16d, 16e, 18a, 18c, 19b, 19e, 19g, 20b, 20c, 20e, 21b, 21c, 21e, 21h, 21i, 21j, 22e, 25a, 25b, 25c,

25d, 25e, 25f, 25g, 25h, 25i, 25j, 27, 28, 29, 32, 34, 35a, 35b, 35c, 35d, 36d, 36h, 36i, and 36j and remove the remaining columns, then we have 245 rows, 49 columns and 2405 missing values. This subdataset is called *US-FD1W-25(d)*.

Data Description.

To have a better understanding of questions (variables) of the data set [?], we categorize them into ten categories as follows:

- Demographic Information: Questions 1-8.
- Academic Life: Questions 9-18 & 20.
- Satisfaction with University, Government, and other organizations: Questions 19, 35, 36.
- Access to required materials/skills for online learning: Questions 21 &22.
- Social Life/Support Network: Questions 23 & 24.
- Emotions: Question 25.
- Worries/concerns: Question 26.
- Finances: Questions 27 – 34.
- Forced move due to COVID: Question 37.
- Habits/Choices before and since the pandemic: Question 28.

A list of questions, and their descriptions is given below.

Name	Label	Values
Q1	In which country do you study (in this semester)?	
Q3	Are you a citizen of the country in which you study?	1: "Yes", 2: "No"
Q4	What is your student status?	1: "Full-time", 2: "Part-time"
Q5	What level of study you are enrolled in?	1: "Bachelor's degree", 2: "Master's degree", 3: "Doctoral degree"
Q6	Please indicate your main field of study.	1: "Arts", 2: "Social", 3: "Natural", 4: "Techincal"
Q7	How old are you (in years)? (Click and write)	
Q8	What is your gender?	1: "Male", 2: "Female", 3: "Gender diverse", 4:"Prefer not to say"
Q9	Have your on-site classes (those taking place in the location/campus of your study institution) been cancelled due to the COVID-19 pandemic?	1: "Yes", 2: "No"
Q10	Since on-site classes were cancelled, the organization of lectures has changed. Below, several different forms of online lectures are listed. Please assess your level of satisfaction with each form. If you have no experience with particular form, please select "Not applicable".	
Q10a	Online in real-time (videoconference)	1: "Very dissatisfied", 2: "Dissatisfied", 3: "Natural", 4: "Satisfied", 5: "Very satisfied"
Q10b	Online with a video recording (not in real-time)	1: "Very dissatisfied", 2: "Dissatisfied", 3: "Natural", 4: "Satisfied", 5: "Very satisfied"
Q10c	Online with an audio recording (not in real-time)	1: "Very dissatisfied", 2: "Dissatisfied", 3: "Natural", 4: "Satisfied", 5: "Very satisfied"
Q10d	Online by sending presentations to students	1: "Very dissatisfied", 2: "Dissatisfied", 3: "Natural", 4: "Satisfied", 5: "Very satisfied"
Q10e	Written communication (forums, chat, etc.)	1: "Very dissatisfied", 2: "Dissatisfied", 3: "Natural", 4: "Satisfied", 5: "Very satisfied"

Name	Label	Values
Q11	Which of these forms of online lectures has been the most dominant?	1: "Online in real-time (videoconference)", 2: "Online with a video recording (not in real-time)", 3: "Online with an audio recording (not in real-time)", 4: "Online by sending presentations to students", 5: "Written communication (forums, chat, etc.)"
Q12	Since on-site classes were cancelled, the organization of tutorials/seminars and practical classes has changed. Below, several different forms of online tutorials/seminars and practical classes are listed. Please assess your level of satisfaction with each form. If you have no experience with particular form, please select "Not applicable".	
Q12a	Online in real-time (videoconference)	1: "Very dissatisfied", 2: "Dissatisfied", 3: "Natural", 4: "Satisfied", 5: "Very satisfied"
Q12b	Online with a video recording (not in real-time)	1: "Very dissatisfied", 2: "Dissatisfied", 3: "Natural", 4: "Satisfied", 5: "Very satisfied"
Q12c	Online with an audio recording (not in real-time)	1: "Very dissatisfied", 2: "Dissatisfied", 3: "Natural", 4: "Satisfied", 5: "Very satisfied"
Q12d	Online by sending presentations to students	1: "Very dissatisfied", 2: "Dissatisfied", 3: "Natural", 4: "Satisfied", 5: "Very satisfied"
Q12e	Written communication (forums, chat, etc.)	1: "Very dissatisfied", 2: "Dissatisfied", 3: "Natural", 4: "Satisfied", 5: "Very satisfied"
Q13	Which of these forms of online tutorials/seminars and practical classes has been the most dominant?	1: "Online in real-time (videoconference)", 2: "Online with a video recording (not in real-time)", 3: "Online with an audio recording (not in real-time)", 4: "Online by sending presentations to students", 5: "Written communication (forums, chat, etc.)"

Name	Label	Values
Q14	Since on-site classes were cancelled, my supervisions/mentorship has been organized (please select ALL the answers that are true for you).	
Q14a	Via video-call	1: "Yes", 0: "No"
Q14b	Via voice-call	1: "Yes", 0: "No"
Q14c	Via e-mail communication	1: "Yes", 0: "No"
Q14d	Via texting on social networks (Facebook messenger, Viber, WhatsApp, WeChat, etc.)	1: "Yes", 0: "No"
Q14e	Not applicable (I had no supervisions/mentorships)	1: "Yes", 0: "No"
Q15	Which is your preferred method of online supervisions/mentorship?	1: "Via video-call", 2: "Via voice-call", 3: "Via e-mail communication", 4: "Via texting on social networks (Facebook messenger, Viber, WhatsApp, WeChat, etc.)", 5: "Not applicable (I had no supervisions/mentorships)"
Q16	Please rate your agreement with the following statements. Since on-site classes were cancelled, my lecturers:	
Q16a	have provided course assignments (e.g. readings, homework, quizzes) on a regular basis.	1: "Strongly disagree", 2: "Disagree", 3: "Natural", 4: "Agree", 5: "Strongly agree"
Q16b	have provided feedback on my performance on given assignments.	1: "Strongly disagree", 2: "Disagree", 3: "Natural", 4: "Agree", 5: "Strongly agree"
Q16c	have responded to my questions in a timely manner.	1: "Strongly disagree", 2: "Disagree", 3: "Natural", 4: "Agree", 5: "Strongly agree"
Q16d	have been open to students' suggestions and adjustments of online classes.	1: "Strongly disagree", 2: "Disagree", 3: "Natural", 4: "Agree", 5: "Strongly agree"
Q16e	have informed me on what exams will look like in this new situation.	1: "Strongly disagree", 2: "Disagree", 3: "Natural", 4: "Agree", 5: "Strongly agree"
Q17	On average, compared to the workload before on-site classes were cancelled, would you say that your study workload over the last weeks has been:	1: "Significantly smaller", 2: "Smaller", 3: "The same", 4: "Larger", 5: "Significantly larger"

Name	Label	Values
Q18	Since on-site classes were cancelled, how satisfied have you been with the organization of:	
Q18a	lectures.	1: "Very dissatisfied", 2: "Dissatisfied", 3: "Natural", 4: "Satisfied", 5: "Very satisfied"
Q18b	tutorials/seminars and practical classes.	1: "Very dissatisfied", 2: "Dissatisfied", 3: "Natural", 4: "Satisfied", 5: "Very satisfied"
Q18c	supervisions (mentorships).	1: "Very dissatisfied", 2: "Dissatisfied", 3: "Natural", 4: "Satisfied", 5: "Very satisfied"
Q19	Since on-site classes were cancelled, how satisfied have you been with support of:	
Q19a	teaching staff.	1: "Very dissatisfied", 2: "Dissatisfied", 3: "Natural", 4: "Satisfied", 5: "Very satisfied"
Q19b	technical support or IT services.	1: "Very dissatisfied", 2: "Dissatisfied", 3: "Natural", 4: "Satisfied", 5: "Very satisfied"
Q19c	student affairs office.	1: "Very dissatisfied", 2: "Dissatisfied", 3: "Natural", 4: "Satisfied", 5: "Very satisfied"
Q19d	finance and accounting.	1: "Very dissatisfied", 2: "Dissatisfied", 3: "Natural", 4: "Satisfied", 5: "Very satisfied"
Q19e	international office.	1: "Very dissatisfied", 2: "Dissatisfied", 3: "Natural", 4: "Satisfied", 5: "Very satisfied"
Q19f	library.	1: "Very dissatisfied", 2: "Dissatisfied", 3: "Natural", 4: "Satisfied", 5: "Very satisfied"
Q19g	public relations (websites and social media information).	1: "Very dissatisfied", 2: "Dissatisfied", 3: "Natural", 4: "Satisfied", 5: "Very satisfied"
Q19h	tutors.	1: "Very dissatisfied", 2: "Dissatisfied", 3: "Natural", 4: "Satisfied", 5: "Very satisfied"

Name	Label	Values
Q19i	student counseling services.	1: "Very dissatisfied", 2: "Dissatisfied", 3: "Natural", 4: "Satisfied", 5: "Very satisfied"
Q20	In view of the new teaching and learning environment, to what extent do you agree with the following statements.	
Q20a	It is more difficult for me to focus during online teaching in comparison to on-site teaching.	1: "Strongly disagree", 2: "Disagree", 3: "Natural", 4: "Agree", 5: "Strongly agree"
Q20b	My performance as a student has improved since on-site classes were cancelled.	1: "Strongly disagree", 2: "Disagree", 3: "Natural", 4: "Agree", 5: "Strongly agree"
Q20c	My performance as a student has worsen since on-site classes were cancelled.	1: "Strongly disagree", 2: "Disagree", 3: "Natural", 4: "Agree", 5: "Strongly agree"
Q20d	I have adapted well to the new teaching and learning experience.	1: "Strongly disagree", 2: "Disagree", 3: "Natural", 4: "Agree", 5: "Strongly agree"
Q20e	I can master the skills taught in class this year even on-site classes were cancelled.	1: "Strongly disagree", 2: "Disagree", 3: "Natural", 4: "Agree", 5: "Strongly agree"
Q20f	I can figure out how to do the most difficult classwork since on-site classes were cancelled.	1: "Strongly disagree", 2: "Disagree", 3: "Natural", 4: "Agree", 5: "Strongly agree"
Q21	In your home, do you have access to the following?	
Q21a	A quiet place to study	1: "Never", 2: "Rarely", 3: "Sometimes", 4: "Often", 5: "Always"
Q21b	A desk	1: "Never", 2: "Rarely", 3: "Sometimes", 4: "Often", 5: "Always"
Q21c	A computer	1: "Never", 2: "Rarely", 3: "Sometimes", 4: "Often", 5: "Always"
Q21d	Required software and programmes	1: "Never", 2: "Rarely", 3: "Sometimes", 4: "Often", 5: "Always"

Name	Label	Values
Q21e	A printer	1: "Never", 2: "Rarely", 3: "Sometimes", 4: "Often", 5: "Always"
Q21f	Headphones and microphone	1: "Never", 2: "Rarely", 3: "Sometimes", 4: "Often", 5: "Always"
Q21g	Webcam	1: "Never", 2: "Rarely", 3: "Sometimes", 4: "Often", 5: "Always"
Q21h	Office supplies (notebooks, pens, etc.)	1: "Never", 2: "Rarely", 3: "Sometimes", 4: "Often", 5: "Always"
Q21i	A good internet connection	1: "Never", 2: "Rarely", 3: "Sometimes", 4: "Often", 5: "Always"
Q21j	Course study material (e.g. compulsory and recommended literature)	1: "Never", 2: "Rarely", 3: "Sometimes", 4: "Often", 5: "Always"
Q22	To what extent do you agree with the following statements about your computer skills. I am confident in:	
Q22a	browsing online information.	1: "Strongly disagree", 2: "Disagree", 3: "Natural", 4: "Agree", 5: "Strongly agree"
Q22b	sharing digital content.	1: "Strongly disagree", 2: "Disagree", 3: "Natural", 4: "Agree", 5: "Strongly agree"
Q22c	using online teaching platforms (BigBlueButton, Moodle, Blackboard, GoToMeeting, etc.).	1: "Strongly disagree", 2: "Disagree", 3: "Natural", 4: "Agree", 5: "Strongly agree"
Q22d	using online collaboration platforms (Zoom, MS Teams, Skype, etc.).	1: "Strongly disagree", 2: "Disagree", 3: "Natural", 4: "Agree", 5: "Strongly agree"
Q22e	using online communication platforms (e-mail, messaging, etc.).	1: "Strongly disagree", 2: "Disagree", 3: "Natural", 4: "Agree", 5: "Strongly agree"
Q22f	using software and programmes required for my studies.	1: "Strongly disagree", 2: "Disagree", 3: "Natural", 4: "Agree", 5: "Strongly agree"
Q22g	applying advanced settings to some software and programmes.	1: "Strongly disagree", 2: "Disagree", 3: "Natural", 4: "Agree", 5: "Strongly agree"

Name	Label	Values
Q23	How often have you communicated with the following people online since the COVID-19 pandemic online?	
Q23a	Close family member	1: "Not at all", 2: "Two or three times a month", 3: "Once a week", 4: "Several times a week", 5: "Once a day", 6: "Several times a day"
Q23b	More distant family member	1: "Not at all", 2: "Two or three times a month", 3: "Once a week", 4: "Several times a week", 5: "Once a day", 6: "Several times a day"
Q23c	Close friend	1: "Not at all", 2: "Two or three times a month", 3: "Once a week", 4: "Several times a week", 5: "Once a day", 6: "Several times a day"
Q23d	Someone I live with (e.g. roommate)	1: "Not at all", 2: "Two or three times a month", 3: "Once a week", 4: "Several times a week", 5: "Once a day", 6: "Several times a day"
Q23e	Neighbours	1: "Not at all", 2: "Two or three times a month", 3: "Once a week", 4: "Several times a week", 5: "Once a day", 6: "Several times a day"
Q23f	Colleague from my course	1: "Not at all", 2: "Two or three times a month", 3: "Once a week", 4: "Several times a week", 5: "Once a day", 6: "Several times a day"
Q23g	Lecturer	1: "Not at all", 2: "Two or three times a month", 3: "Once a week", 4: "Several times a week", 5: "Once a day", 6: "Several times a day"
Q23h	Administrative staff	1: "Not at all", 2: "Two or three times a month", 3: "Once a week", 4: "Several times a week", 5: "Once a day", 6: "Several times a day"

Name	Label	Values
Q23i	Voluntary organizations	1: "Not at all", 2: "Two or three times a month", 3: "Once a week", 4: "Several times a week", 5: "Once a day", 6: "Several times a day"
Q23j	Social networks	1: "Not at all", 2: "Two or three times a month", 3: "Once a week", 4: "Several times a week", 5: "Once a day", 6: "Several times a day"
Q24	For each of the following situations, please select who you would turn to first. If there are several people you are equally likely to turn to, please select the one you feel closest to. Who would you turn to first to?	
Q24a	Help you around your home if you were sick and had to stay in bed for a few days.	1: "Close family member", 2: "More distant family member", 3: "Close friend", 4: "Someone I live with (e.g. roommate)", 5: "Neighbours", 6: "Colleague from my course", 7: "Lecturer", 8: "Administrative staff", 9: "Voluntary organizations", 10: "Social networks", 12: "No one"
Q24b	Be there for you if you felt a bit down or depressed and wanted to talk about it.	1: "Close family member", 2: "More distant family member", 3: "Close friend", 4: "Someone I live with (e.g. roommate)", 5: "Neighbours", 6: "Colleague from my course", 7: "Lecturer", 8: "Administrative staff", 9: "Voluntary organizations", 10: "Social networks", 12: "No one"
Q24c	Talk about problems related to studying issues (lectures, seminars, practical work).	1: "Close family member", 2: "More distant family member", 3: "Close friend", 4: "Someone I live with (e.g. roommate)", 5: "Neighbours", 6: "Colleague from my course", 7: "Lecturer", 8: "Administrative staff", 9: "Voluntary organizations", 10: "Social networks", 12: "No one"

Name	Label	Values
Q24d	Talk about problems related to future education.	1: "Close family member", 2: "More distant family member", 3: "Close friend", 4: "Someone I live with (e.g. roommate)", 5: "Neighbours", 6: "Colleague from my course", 7: "Lecturer", 8: "Administrative staff", 9: "Voluntary organizations", 10: "Social networks", 12: "No one"
Q24e	Talk about problems related to personal finances.	1: "Close family member", 2: "More distant family member", 3: "Close friend", 4: "Someone I live with (e.g. roommate)", 5: "Neighbours", 6: "Colleague from my course", 7: "Lecturer", 8: "Administrative staff", 9: "Voluntary organizations", 10: "Social networks", 12: "No one"
Q24f	Talk about problems related to family and relationships.	1: "Close family member", 2: "More distant family member", 3: "Close friend", 4: "Someone I live with (e.g. roommate)", 5: "Neighbours", 6: "Colleague from my course", 7: "Lecturer", 8: "Administrative staff", 9: "Voluntary organizations", 10: "Social networks", 12: "No one"
Q24g	Talk about problems related to professional career in the future.	1: "Close family member", 2: "More distant family member", 3: "Close friend", 4: "Someone I live with (e.g. roommate)", 5: "Neighbours", 6: "Colleague from my course", 7: "Lecturer", 8: "Administrative staff", 9: "Voluntary organizations", 10: "Social networks", 12: "No one"

Name	Label	Values
Q24h	Be there for you if you would like to talk about the COVID-19 crisis.	1: "Close family member", 2: "More distant family member", 3: "Close friend", 4: "Someone I live with (e.g. roommate)", 5: "Neighbours", 6: "Colleague from my course", 7: "Lecturer", 8: "Administrative staff", 9: "Voluntary organizations", 10: "Social networks", 12: "No one"
Q25	Please rate to what extent have you felt the following emotions while attending your classes and studying and preparing for them since the outbreak of COVID-19 in your country.	
Q25a	Joyful	1: "Never", 2: "Rarely", 3: "Sometimes", 4: "Often", 5: "Always"
Q25b	Hopeful	1: "Never", 2: "Rarely", 3: "Sometimes", 4: "Often", 5: "Always"
Q25c	Proud	1: "Never", 2: "Rarely", 3: "Sometimes", 4: "Often", 5: "Always"
Q25d	Frustrated	1: "Never", 2: "Rarely", 3: "Sometimes", 4: "Often", 5: "Always"
Q25e	Angry	1: "Never", 2: "Rarely", 3: "Sometimes", 4: "Often", 5: "Always"
Q25f	Anxious	1: "Never", 2: "Rarely", 3: "Sometimes", 4: "Often", 5: "Always"
Q25g	Ashamed	1: "Never", 2: "Rarely", 3: "Sometimes", 4: "Often", 5: "Always"
Q25h	Relieved	1: "Never", 2: "Rarely", 3: "Sometimes", 4: "Often", 5: "Always"
Q25i	Hopeless	1: "Never", 2: "Rarely", 3: "Sometimes", 4: "Often", 5: "Always"
Q25j	Bored	1: "Never", 2: "Rarely", 3: "Sometimes", 4: "Often", 5: "Always"

Name	Label	Values
Q26	How often you have worries about the following personal circumstances?	
Q26a	Personal physical health	1: "A little of the time", 2: "Some of the time", 3: "A good part of the time", 4: "Most of the time", 5: "All of the time"
Q26b	Personal mental health	1: "A little of the time", 2: "Some of the time", 3: "A good part of the time", 4: "Most of the time", 5: "All of the time"
Q26c	Studying issues (lectures, seminars, practical work)	1: "A little of the time", 2: "Some of the time", 3: "A good part of the time", 4: "Most of the time", 5: "All of the time"
Q26d	Future education	1: "A little of the time", 2: "Some of the time", 3: "A good part of the time", 4: "Most of the time", 5: "All of the time"
Q26e	Personal finances	1: "A little of the time", 2: "Some of the time", 3: "A good part of the time", 4: "Most of the time", 5: "All of the time"
Q26f	Family and relationship	1: "A little of the time", 2: "Some of the time", 3: "A good part of the time", 4: "Most of the time", 5: "All of the time"
Q26g	Professional career in the future	1: "A little of the time", 2: "Some of the time", 3: "A good part of the time", 4: "Most of the time", 5: "All of the time"
Q26h	COVID-19 or similar pandemic crisis in the future	1: "A little of the time", 2: "Some of the time", 3: "A good part of the time", 4: "Most of the time", 5: "All of the time"
Q26i	Leisure activities (sports and cultural activities, parties, hanging out with friends, etc.)	1: "A little of the time", 2: "Some of the time", 3: "A good part of the time", 4: "Most of the time", 5: "All of the time"
Q26j	Traveling abroad	1: "A little of the time", 2: "Some of the time", 3: "A good part of the time", 4: "Most of the time", 5: "All of the time"

Name	Label	Values
Q27	When you take into account your total monthly disposable income (before COVID-19 pandemic) would you say that you can pay the overall costs of your study (including living costs such as accommodation etc.)?	1: "Very easily", 2: "Easily", 3: "Quite easily", 4: "With small difficulty", 5: "With difficulty"
Q28	Have you had a paid job during the current academic year or were you planning on having a paid job during the current academic year?	1: "Yes, I worked regularly this academic year.", 2: "Yes, I worked occasionally this academic year.", 3: "No, I have not worked this academic year but was planning to.", 5: "No, I have not worked this academic year and was not planning to work."
Q29	If you have been working or were planning to work, has this paid job been affected by the COVID-19 pandemic:	1: "Yes, I have lost the job permanently.", 2: "Yes, I have lost the job temporarily.", 3: "Yes, I have had a salary cut.", 4: "No, the job ended before the COVID-19 crisis.", 5: "No, I am still working."
Q30	What are your plans for September/October 2020?	1: "Returning to full-time studies.", 2: "Already accepted a full-time job.", 3: "Still seeking a full-time job."
Q31	Do you pay tuition fees?	1: "Yes", 2: "No"
Q32	In the context of the COVID-19 pandemic please indicate whether:	1: "fee payment has remained the same at my institution.", 2: "my institution has reduced the amount of fees which need to be paid this term.", 3: "my institution has cancelled the payment of fees for this term.", 4: "my institution has introduced flexible ways of paying fees this term (e.g. in more instalments)."
Q33	Do you receive a scholarship for your studies?	1: "Yes", 2: "No"
Q34	In the context of the COVID-19 pandemic please indicate whether:	1: "the amount of my scholarship has remained the same.", 2: "the amount of my scholarship has been reduced.", 3: "my scholarship has been cancelled.", 4: "payment of the scholarship has been postponed."

Name	Label	Values
Q35	Please assess the satisfaction in dealing with the COVID-19 pandemic of the following institutions.	
Q35a	Government	1: "Very dissatisfied", 2: "Dissatisfied", 3: "Natural", 4: "Satisfied", 5: "Very satisfied"
Q35b	University	1: "Very dissatisfied", 2: "Dissatisfied", 3: "Natural", 4: "Satisfied", 5: "Very satisfied"
Q35c	Banks	1: "Very dissatisfied", 2: "Dissatisfied", 3: "Natural", 4: "Satisfied", 5: "Very satisfied"
Q35d	Hospitals	1: "Very dissatisfied", 2: "Dissatisfied", 3: "Natural", 4: "Satisfied", 5: "Very satisfied"
Q36	How important are/will/would be the following actions or support measures by your government, university or by your bank to you?	
Q36a	Transport fares and passes are not required.	1: "Not important", 2: "Slightly important", 3: "Moderately important", 4: "Important", 5: "Very importnant"
Q36b	Freezing rents.	1: "Not important", 2: "Slightly important", 3: "Moderately important", 4: "Important", 5: "Very importnant"
Q36c	Financial assistance for renters.	1: "Not important", 2: "Slightly important", 3: "Moderately important", 4: "Important", 5: "Very importnant"
Q36d	Deferred or reduced mortgage payments.	1: "Not important", 2: "Slightly important", 3: "Moderately important", 4: "Important", 5: "Very importnant"
Q36e	Emergency supports for people on income assistance, disability assistance, and low-income seniors.	1: "Not important", 2: "Slightly important", 3: "Moderately important", 4: "Important", 5: "Very importnant"
Q36f	Monthly payments can be deferred up to 90 days and insurance renewals can now be done by phone or email.	1: "Not important", 2: "Slightly important", 3: "Moderately important", 4: "Important", 5: "Very importnant"

Name	Label	Values
Q36g	Delayed tax filing deadline and due date for taxes owed.	1: "Not important", 2: "Slightly important", 3: "Moderately important", 4: "Important", 5: "Very important"
Q36h	Deferred student loan payments.	1: "Not important", 2: "Slightly important", 3: "Moderately important", 4: "Important", 5: "Very important"
Q36i	Emergency childcare for essential workers.	1: "Not important", 2: "Slightly important", 3: "Moderately important", 4: "Important", 5: "Very important"
Q36j	Delayed taxes.	1: "Not important", 2: "Slightly important", 3: "Moderately important", 4: "Important", 5: "Very important"
Q37	Have you moved due to the COVID-19 pandemic (for example from student dorm to family home, etc.)?	1: "Yes", 2: "No"
Q38_1	Please assess the frequency of your habits before and during the COVID-19 pandemic. - Before the COVID-19 pandemic	
Q38a_1	Washing your hands	1: "Never", 2: "Rarely", 3: "Sometimes", 4: "Often", 5: "Always"
Q38b_1	Leaving the house for unnecessary reasons	1: "Never", 2: "Rarely", 3: "Sometimes", 4: "Often", 5: "Always"
Q38c_1	Avoided crowds and large gatherings	1: "Never", 2: "Rarely", 3: "Sometimes", 4: "Often", 5: "Always"
Q38d_1	Avoided touching your face	1: "Never", 2: "Rarely", 3: "Sometimes", 4: "Often", 5: "Always"
Q38e_1	Shaking hands	1: "Never", 2: "Rarely", 3: "Sometimes", 4: "Often", 5: "Always"

Name	Label	Values
Q38f_1	Stocked up on essentials at pharmacy and grocery store	1: "Never", 2: "Rarely", 3: "Sometimes", 4: "Often", 5: "Always"
Q38g_1	Made a plan for communicating with family friends and neighbours	1: "Never", 2: "Rarely", 3: "Sometimes", 4: "Often", 5: "Always"
Q38h_1	Cancelled travel	1: "Never", 2: "Rarely", 3: "Sometimes", 4: "Often", 5: "Always"
Q38i_1	Filled prescriptions	1: "Never", 2: "Rarely", 3: "Sometimes", 4: "Often", 5: "Always"
Q38j_1	Worked from home	1: "Never", 2: "Rarely", 3: "Sometimes", 4: "Often", 5: "Always"
Q38k_1	Avoiding public transport	1: "Never", 2: "Rarely", 3: "Sometimes", 4: "Often", 5: "Always"
Q38l_1	Wearing a mask outside	1: "Never", 2: "Rarely", 3: "Sometimes", 4: "Often", 5: "Always"
Q38m_1	Offering help to people	1: "Never", 2: "Rarely", 3: "Sometimes", 4: "Often", 5: "Always"
Q38n_1	Online grocery shopping	1: "Never", 2: "Rarely", 3: "Sometimes", 4: "Often", 5: "Always"
Q38o_1	Recreation or workout	1: "Never", 2: "Rarely", 3: "Sometimes", 4: "Often", 5: "Always"
Q38p_1	Visiting family members or friends	1: "Never", 2: "Rarely", 3: "Sometimes", 4: "Often", 5: "Always"

Name	Label	Values
Q38_2	Please assess the frequency of your habits before and during the COVID-19 pandemic. - During the COVID-19 pandemic	
Q38a_2	Washing your hands	1: "Never", 2: "Rarely", 3: "Sometimes", 4: "Often", 5: "Always"
Q38b_2	Leaving the house for unnecessary reasons	1: "Never", 2: "Rarely", 3: "Sometimes", 4: "Often", 5: "Always"
Q38c_2	Avoided crowds and large gatherings	1: "Never", 2: "Rarely", 3: "Sometimes", 4: "Often", 5: "Always"
Q38d_2	Avoided touching your face	1: "Never", 2: "Rarely", 3: "Sometimes", 4: "Often", 5: "Always"
Q38e_2	Shaking hands	1: "Never", 2: "Rarely", 3: "Sometimes", 4: "Often", 5: "Always"
Q38f_2	Stocked up on essentials at pharmacy and grocery store	1: "Never", 2: "Rarely", 3: "Sometimes", 4: "Often", 5: "Always"
Q38g_2	Made a plan for communicating with family friends and neighbours	1: "Never", 2: "Rarely", 3: "Sometimes", 4: "Often", 5: "Always"
Q38h_2	Cancelled travel	1: "Never", 2: "Rarely", 3: "Sometimes", 4: "Often", 5: "Always"
Q38i_2	Filled prescriptions	1: "Never", 2: "Rarely", 3: "Sometimes", 4: "Often", 5: "Always"
Q38j_2	Worked from home	1: "Never", 2: "Rarely", 3: "Sometimes", 4: "Often", 5: "Always"
Q38k_2	Avoiding public transport	1: "Never", 2: "Rarely", 3: "Sometimes", 4: "Often", 5: "Always"
Q38l_2	Wearing a mask outside	1: "Never", 2: "Rarely", 3: "Sometimes", 4: "Often", 5: "Always"
Q38m_2	Offering help to people	1: "Never", 2: "Rarely", 3: "Sometimes", 4: "Often", 5: "Always"
Q38n_2	Online grocery shopping	1: "Never", 2: "Rarely", 3: "Sometimes", 4: "Often", 5: "Always"
Q38o_2	Recreation or workout	1: "Never", 2: "Rarely", 3: "Sometimes", 4: "Often", 5: "Always"
Q38p_2	Visiting family members or friends	1: "Never", 2: "Rarely", 3: "Sometimes", 4: "Often", 5: "Always"
