# Supplementary figures and images for: Artificial intelligence-based analytics for impacts of COVID-19 and online learning on college students’ mental health
Source: PLoS One. 2022 Nov 18;17(11):e0276767. doi: 10.1371/journal.pone.0276767 (PMC9674166; doi:10.1371/journal.pone.0276767)

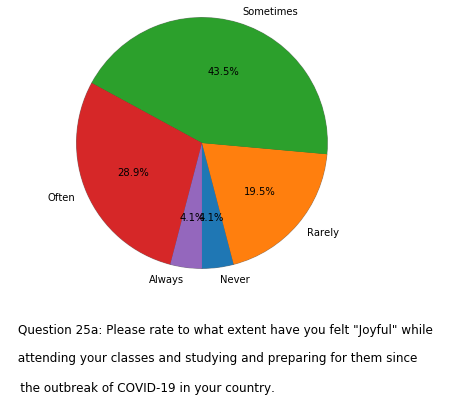

Supplement: S1 Data — (ZIP) [file pone.0276767.s001.zip › Revised/f1.png]

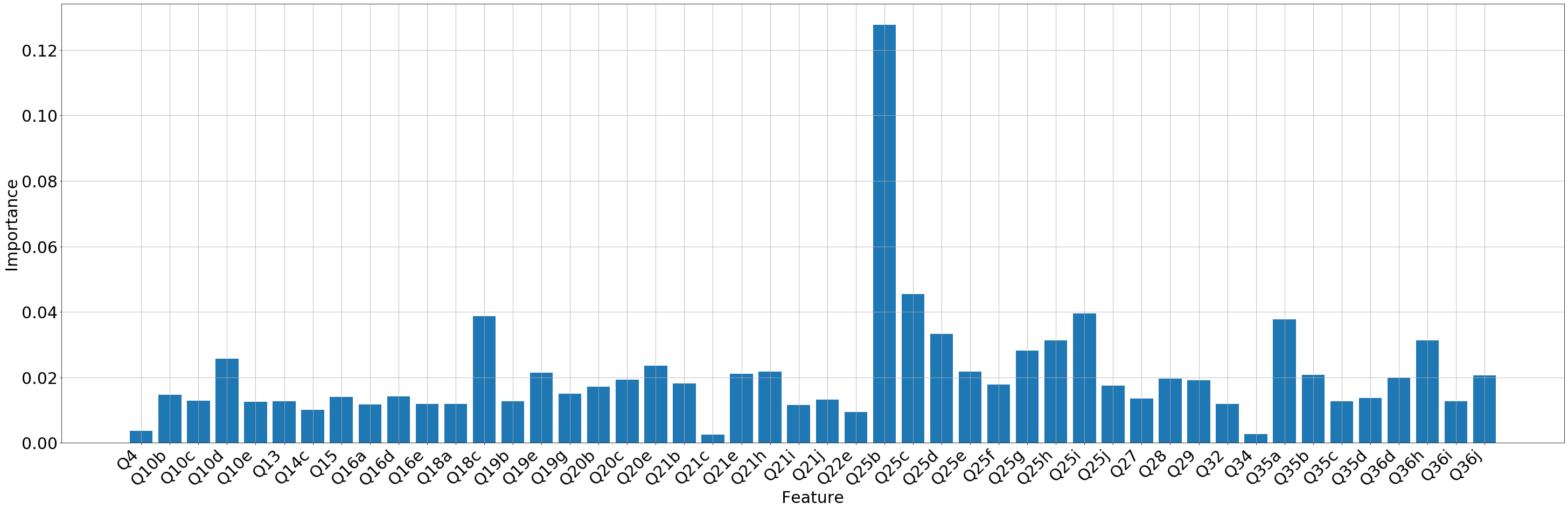

Supplement: S1 Data — (ZIP) [file pone.0276767.s001.zip › Revised/f3.png]

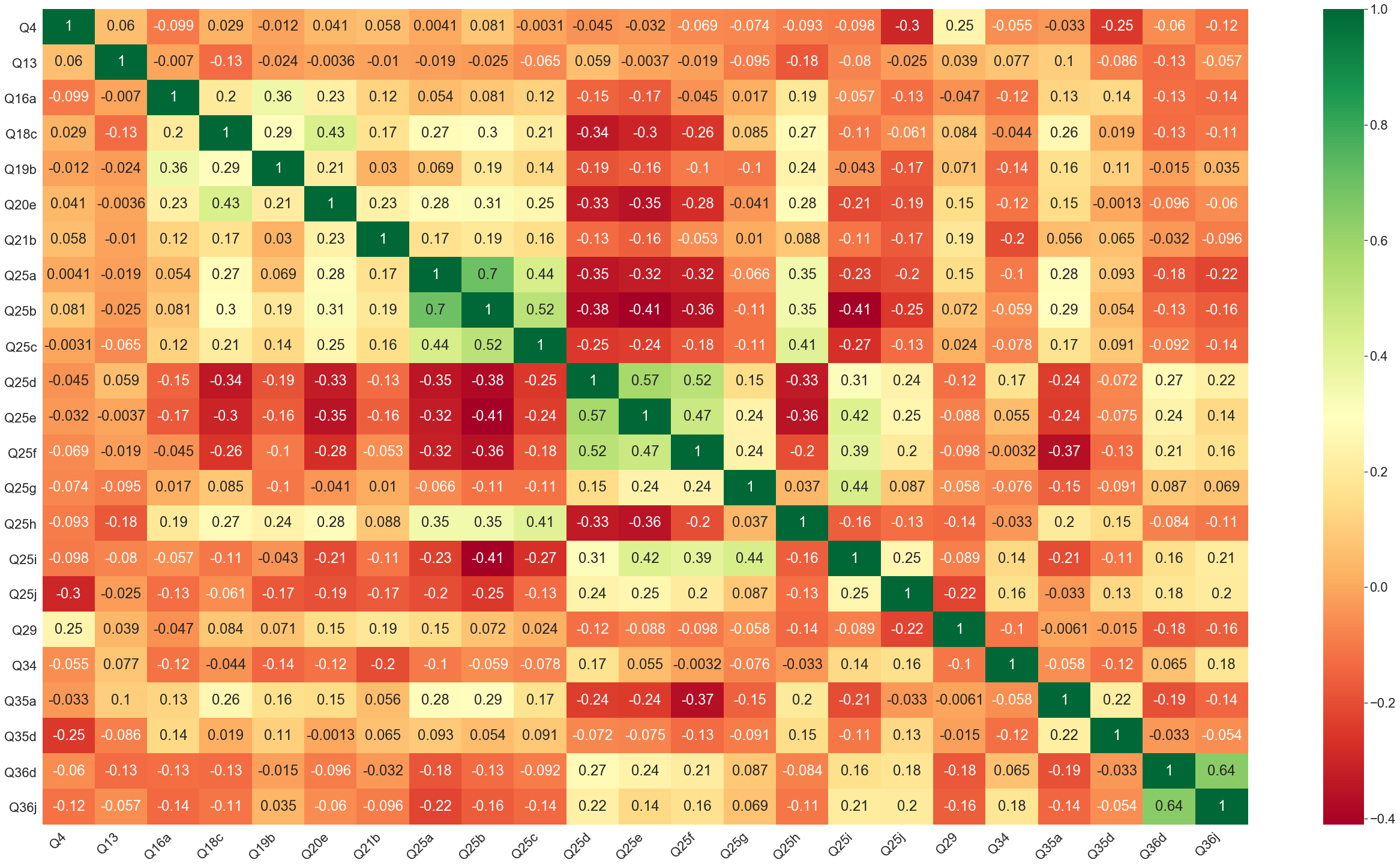

Supplement: S1 Data — (ZIP) [file pone.0276767.s001.zip › Revised/f4.png]

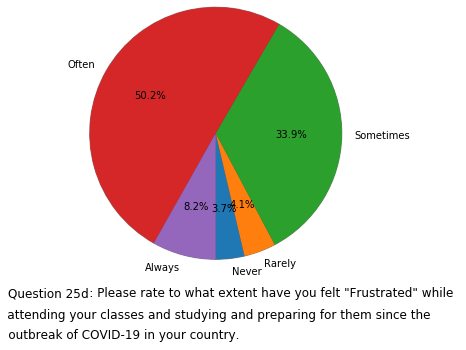

Supplement: S1 Data — (ZIP) [file pone.0276767.s001.zip › Revised/f5.png]

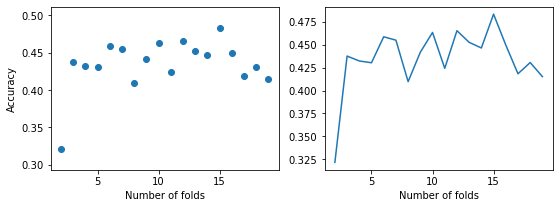

Supplement: S1 Data — (ZIP) [file pone.0276767.s001.zip › Revised/p1.png]

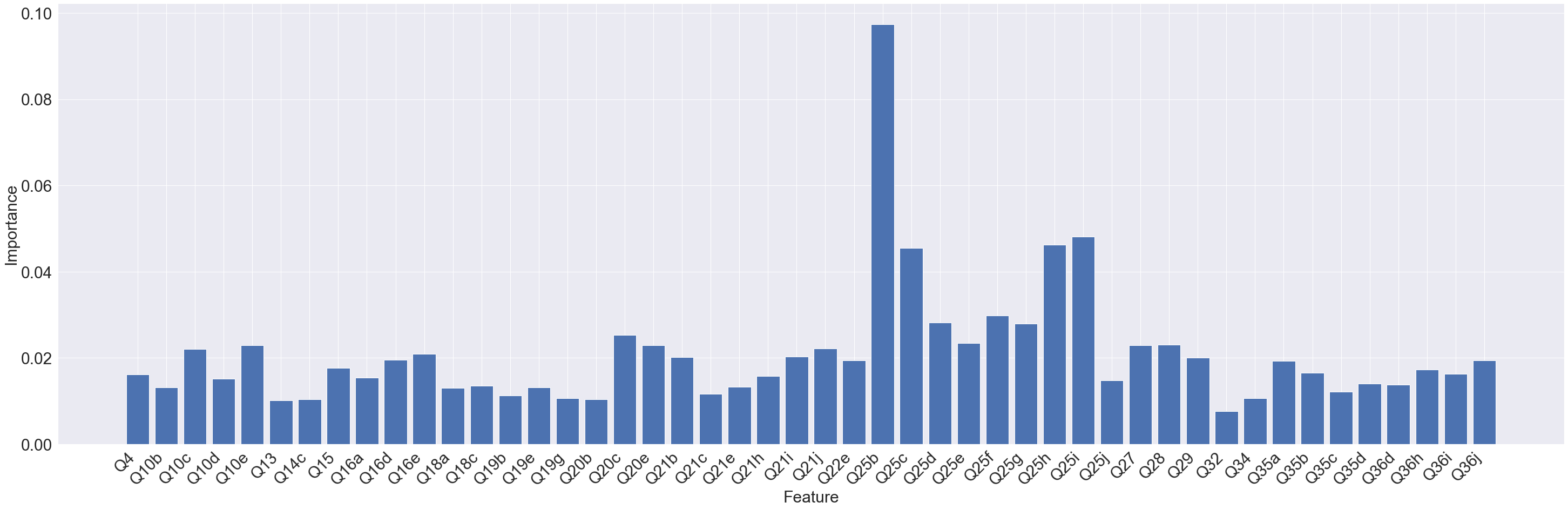

Supplement: S1 Data — (ZIP) [file pone.0276767.s001.zip › Revised/p11.png]

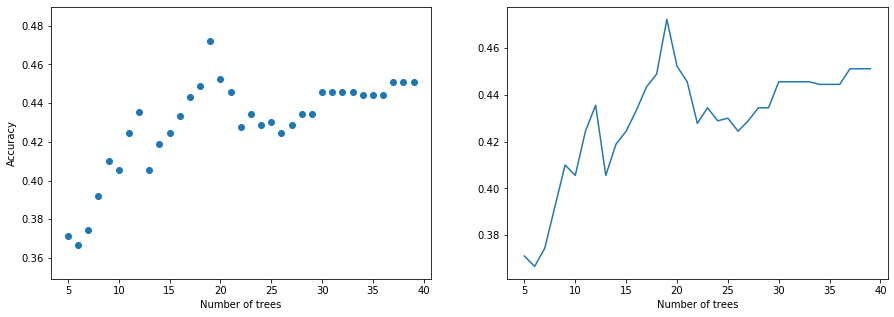

Supplement: S1 Data — (ZIP) [file pone.0276767.s001.zip › Revised/p2.png]

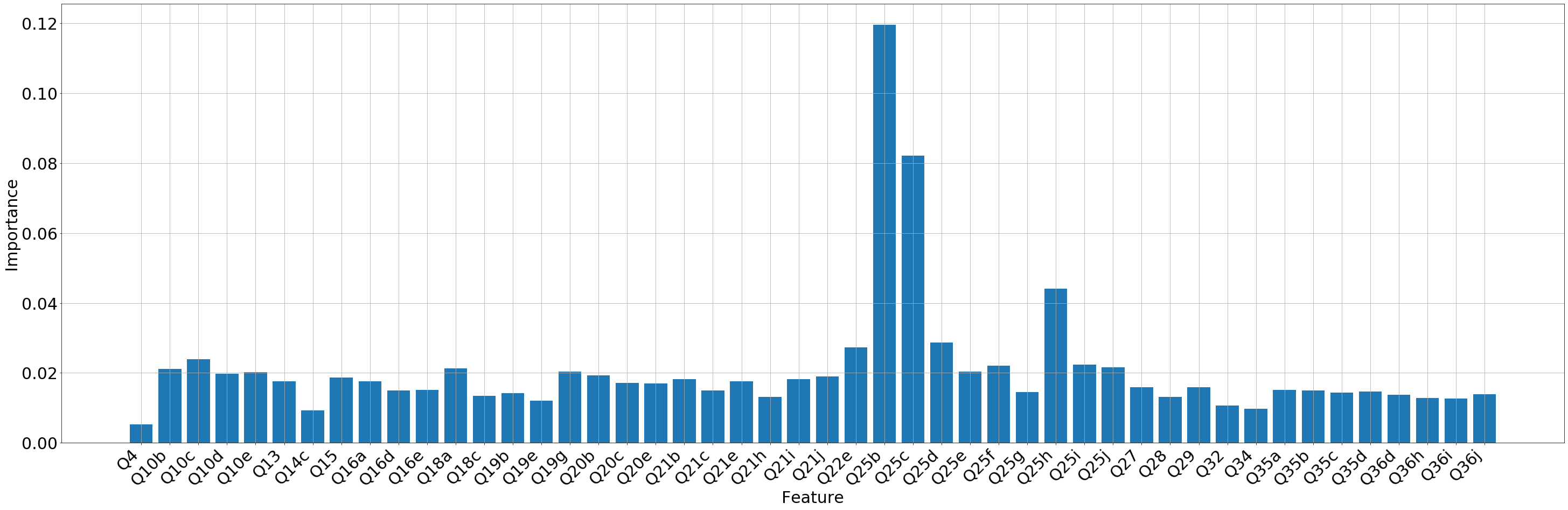

Supplement: S1 Data — (ZIP) [file pone.0276767.s001.zip › Revised/p26.png]

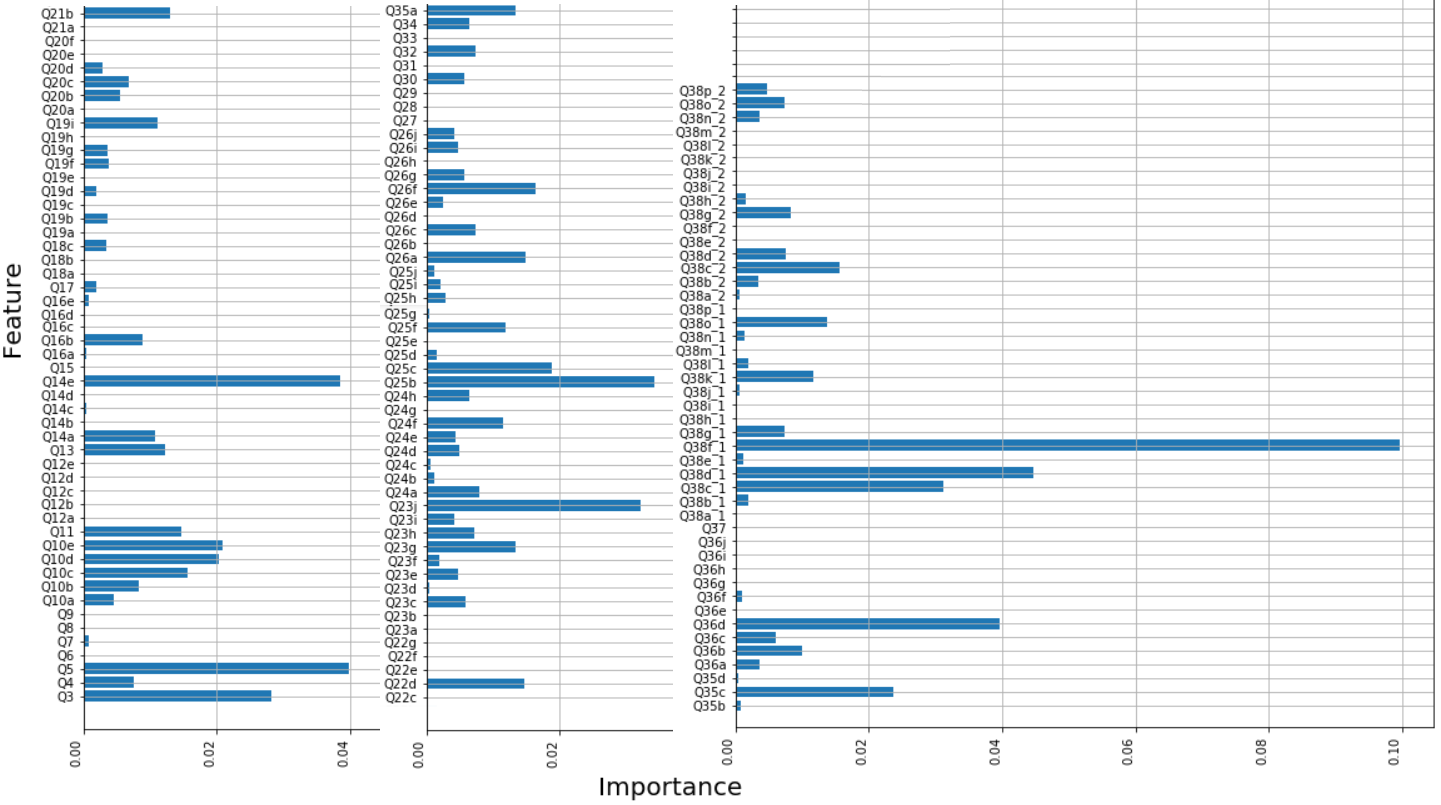

Supplement: S1 Data — (ZIP) [file pone.0276767.s001.zip › Revised/p3.png]

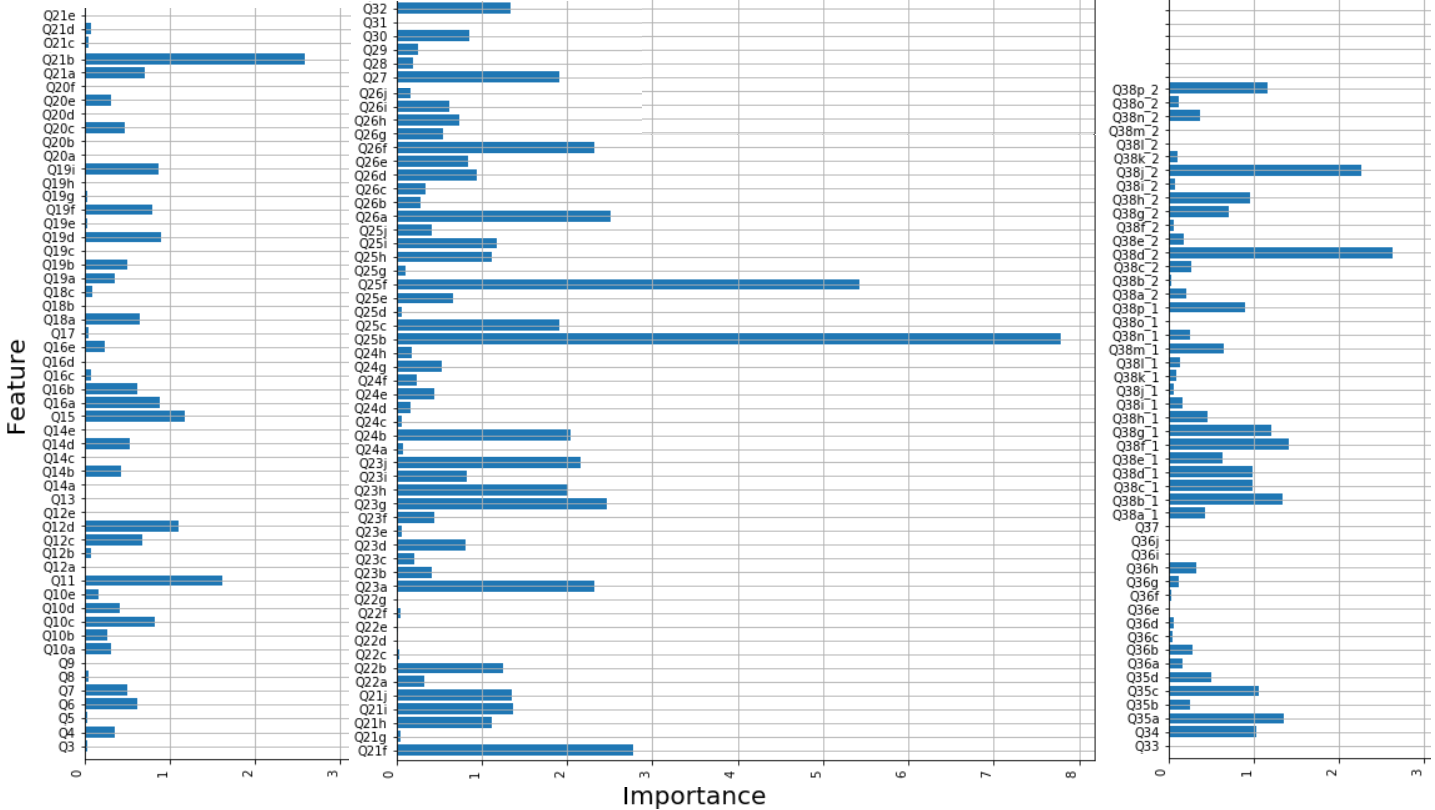

Supplement: S1 Data — (ZIP) [file pone.0276767.s001.zip › Revised/p4.png]

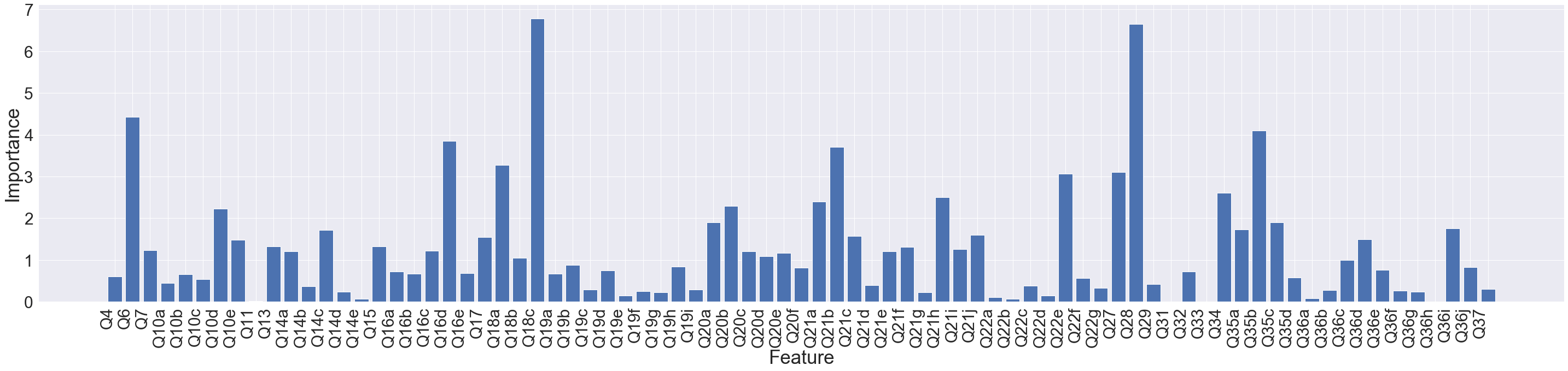

Supplement: S1 Data — (ZIP) [file pone.0276767.s001.zip › Revised/p5.png]

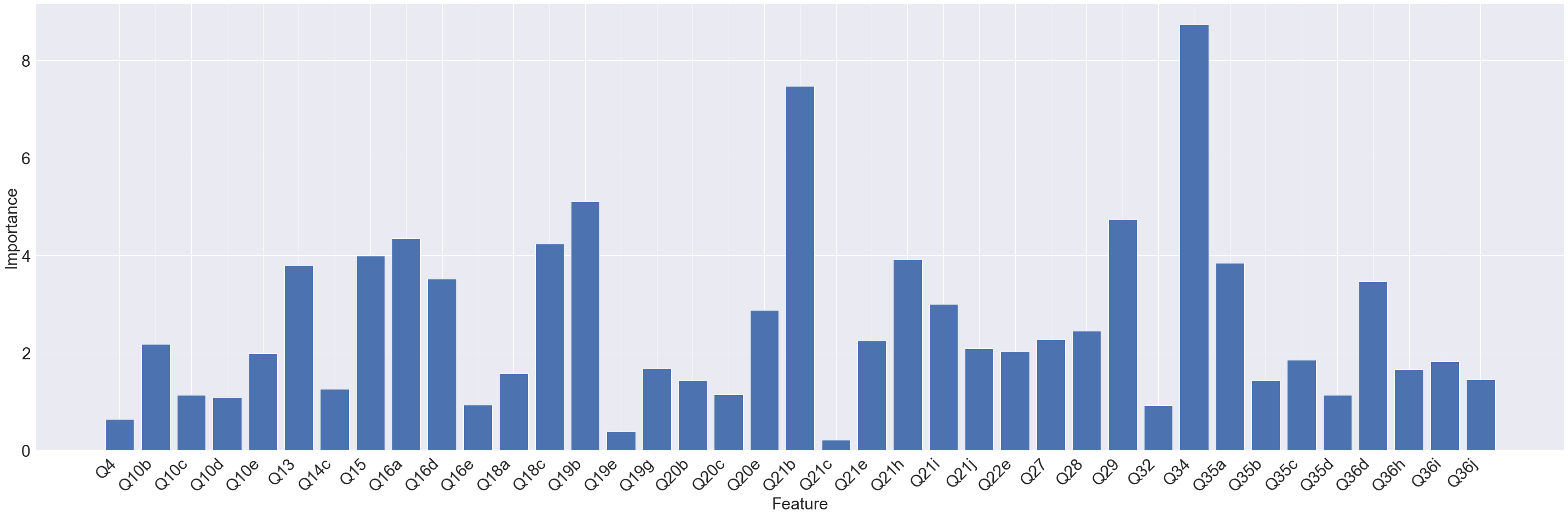

Supplement: S1 Data — (ZIP) [file pone.0276767.s001.zip › Revised/p6.png]

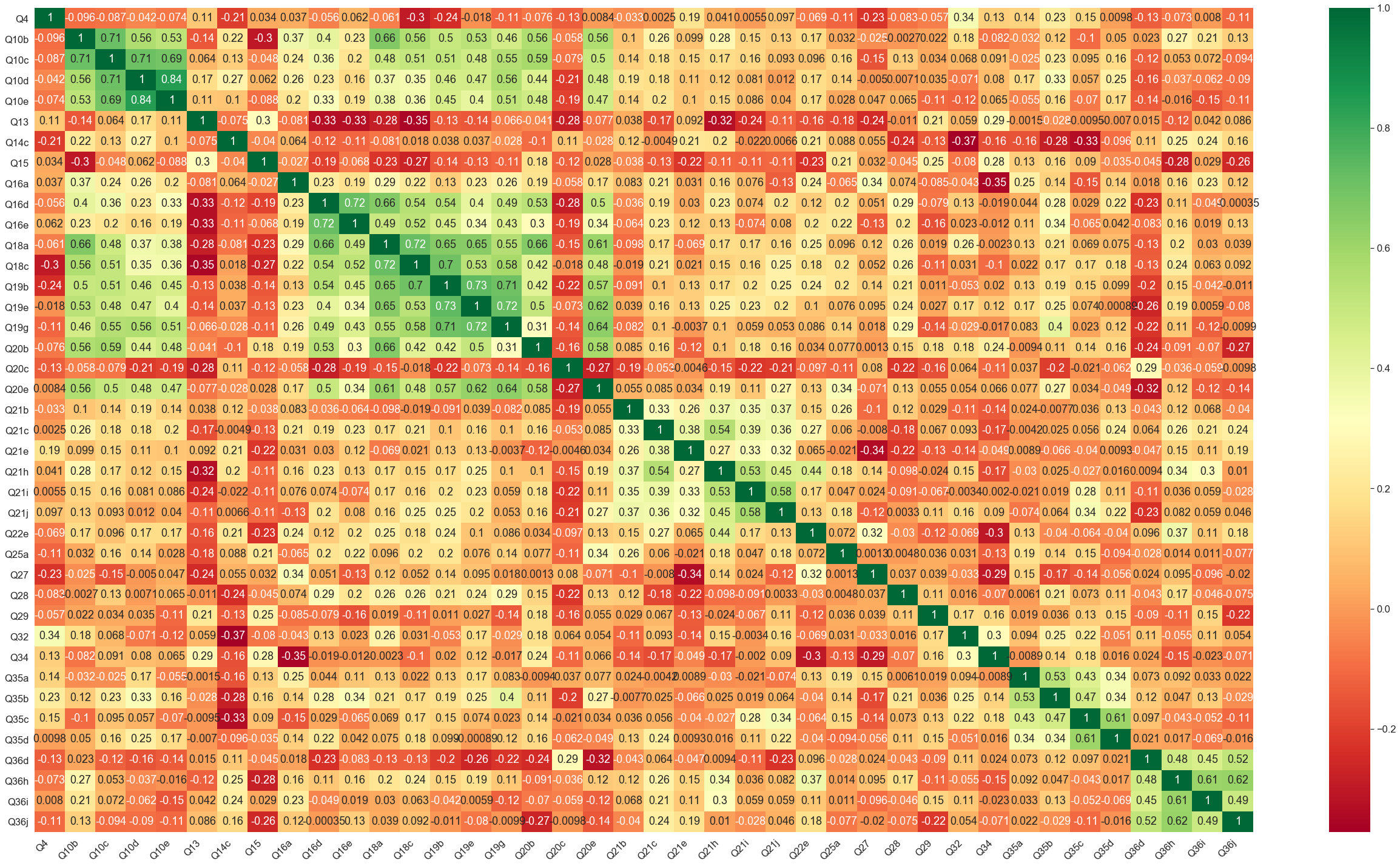

Supplement: S1 Data — (ZIP) [file pone.0276767.s001.zip › Revised/p7.png]

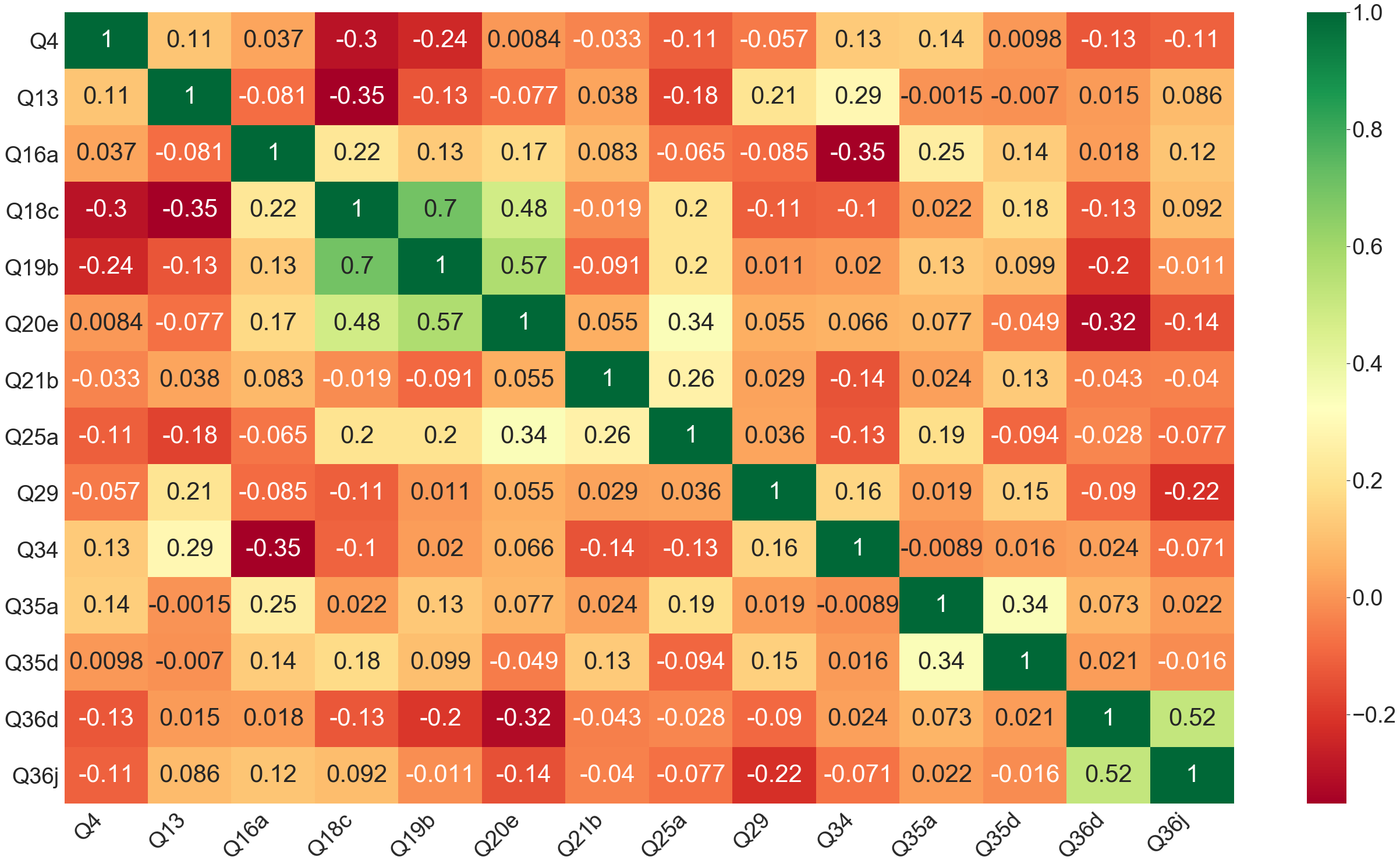

Supplement: S1 Data — (ZIP) [file pone.0276767.s001.zip › Revised/p8.png]

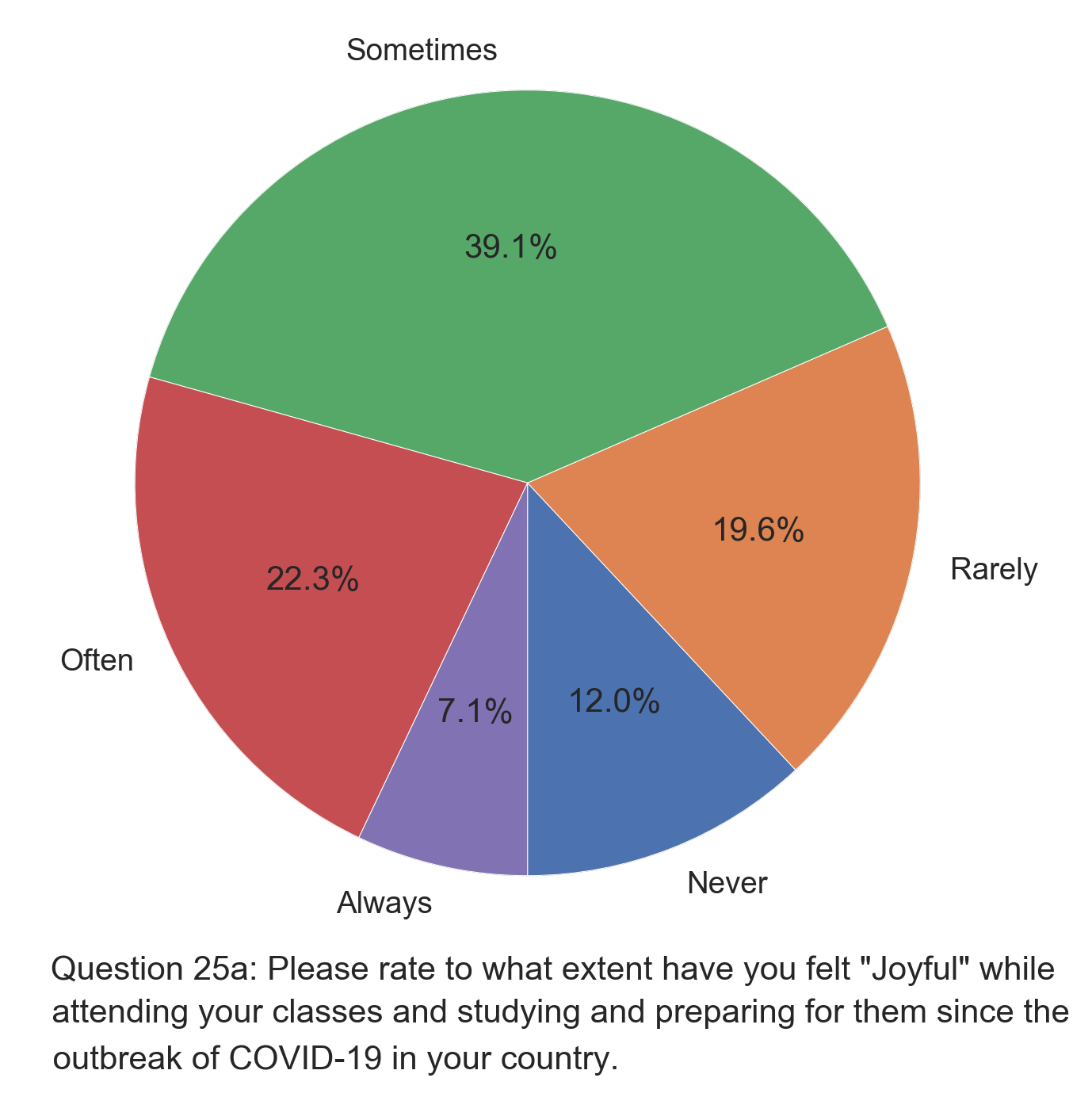

Supplement: S1 Data — (ZIP) [file pone.0276767.s001.zip › Revised/p9.png]

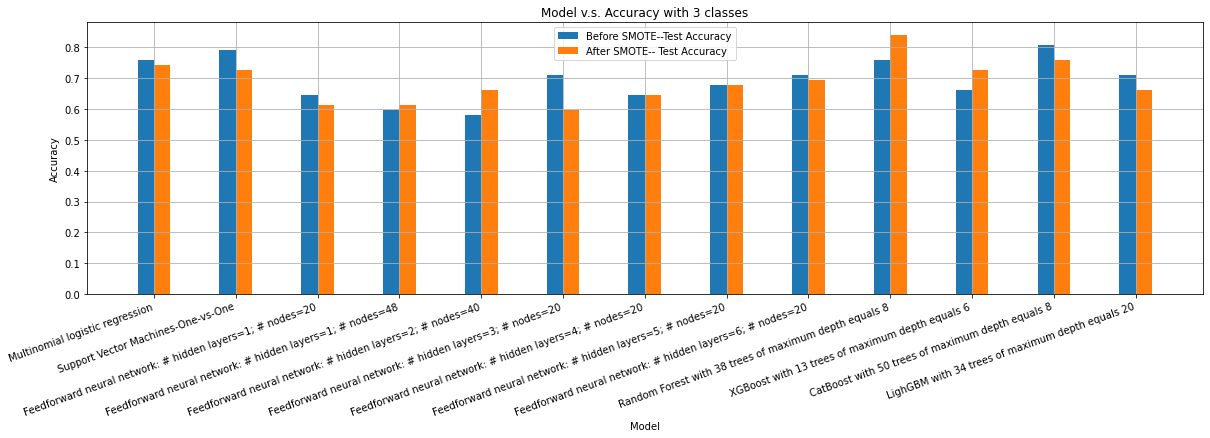

Supplement: S1 Data — (ZIP) [file pone.0276767.s001.zip › Revised/part2-1.png]

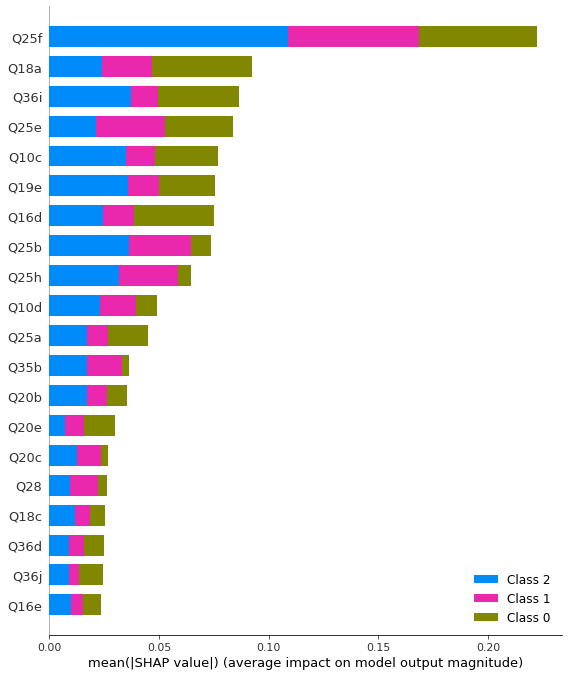

Supplement: S1 Data — (ZIP) [file pone.0276767.s001.zip › Revised/part2-2.png]

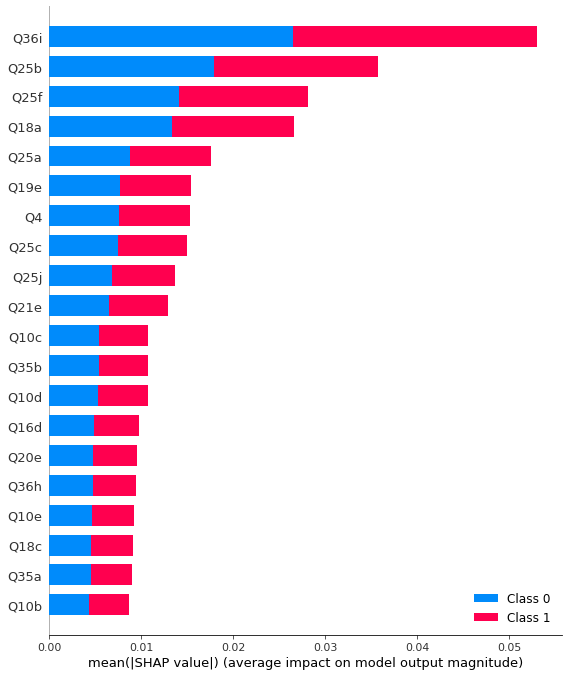

Supplement: S1 Data — (ZIP) [file pone.0276767.s001.zip › Revised/part3-1.png]

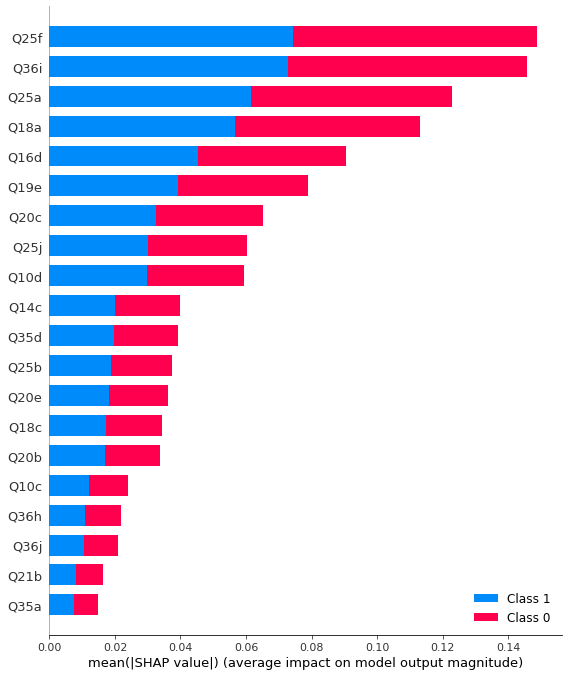

Supplement: S1 Data — (ZIP) [file pone.0276767.s001.zip › Revised/part3-2.png]

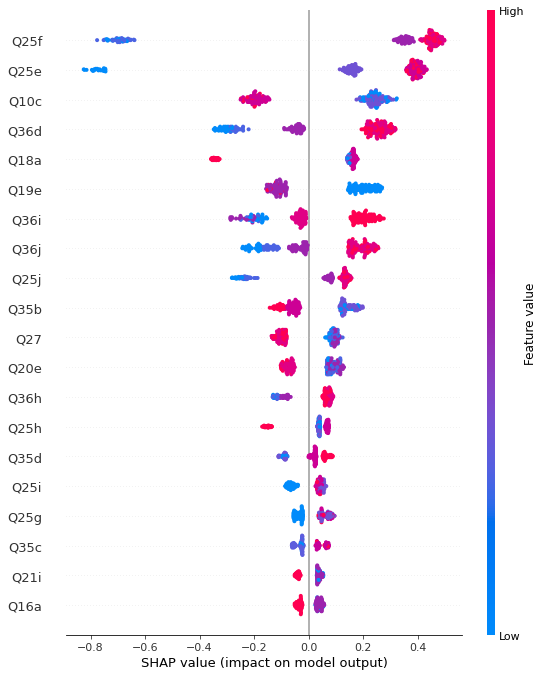

Supplement: S1 Data — (ZIP) [file pone.0276767.s001.zip › Revised/part3-4.png]

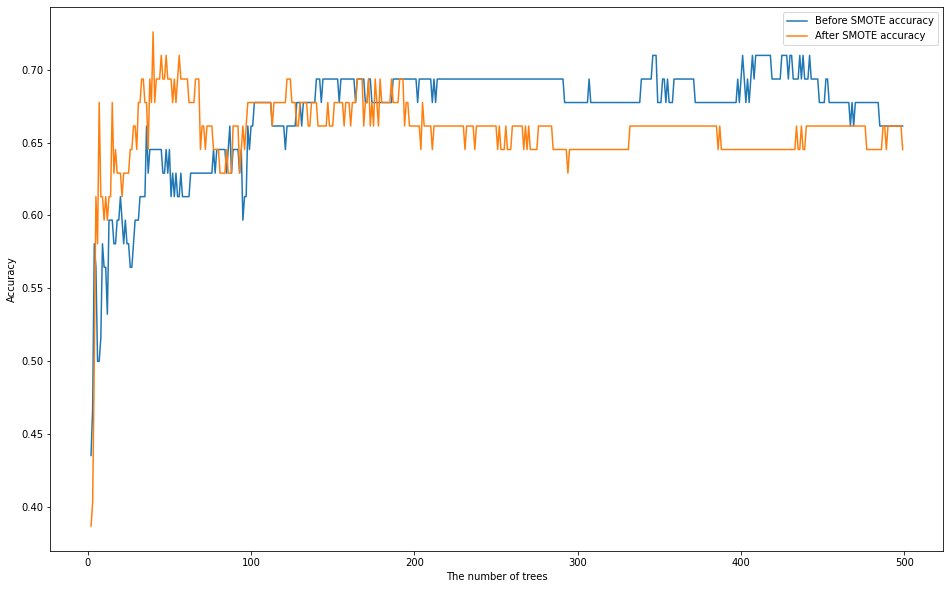

Supplement: S1 Data — (ZIP) [file pone.0276767.s001.zip › Revised/re1.png]

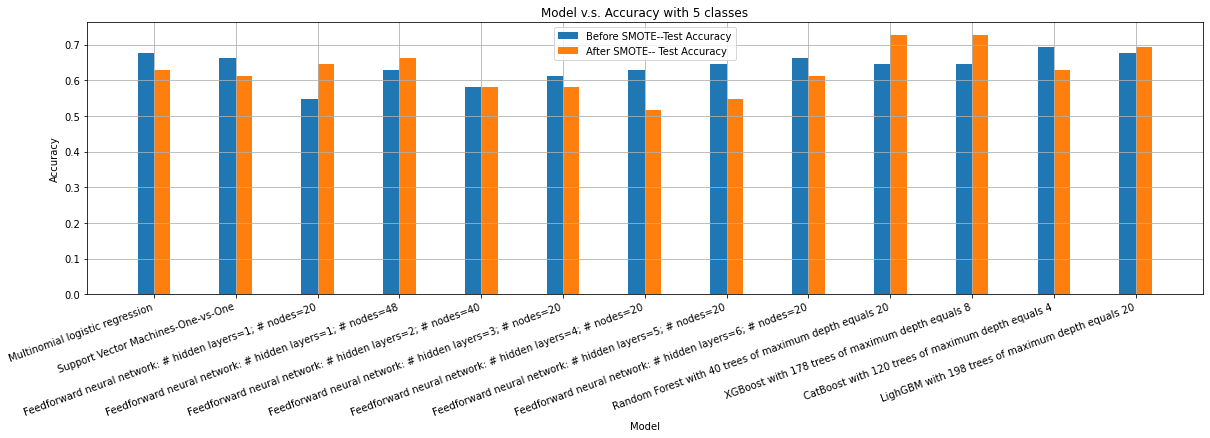

Supplement: S1 Data — (ZIP) [file pone.0276767.s001.zip › Revised/ref10.png]

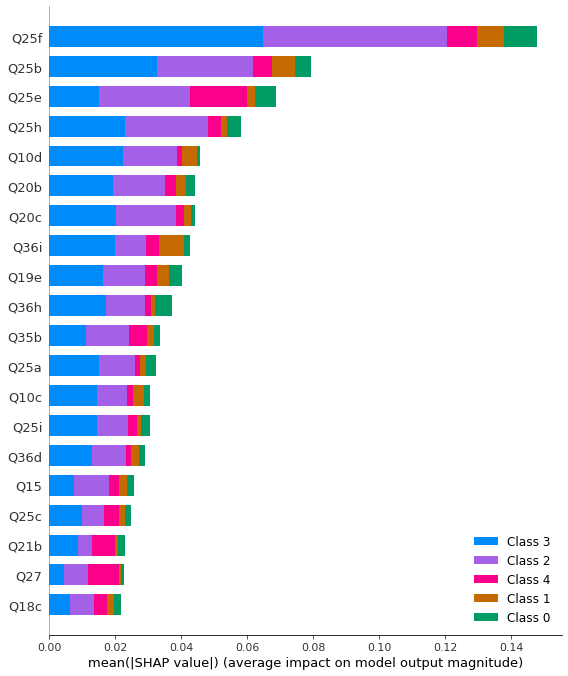

Supplement: S1 Data — (ZIP) [file pone.0276767.s001.zip › Revised/ref2.png]

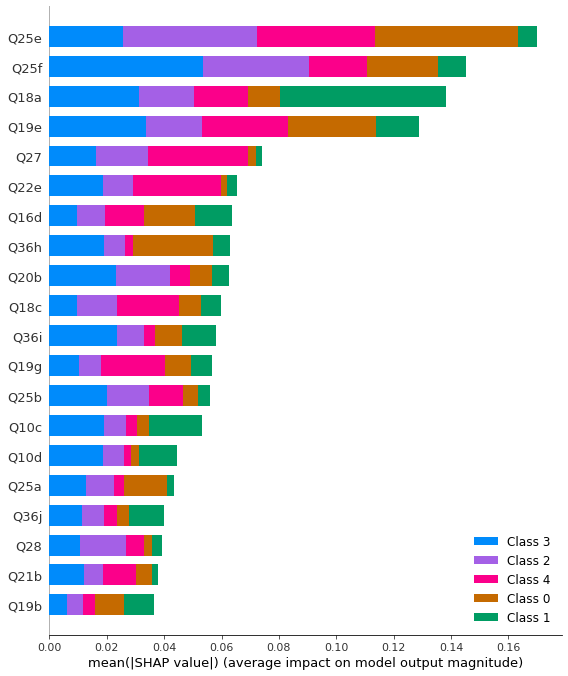

Supplement: S1 Data — (ZIP) [file pone.0276767.s001.zip › Revised/ref3.png]

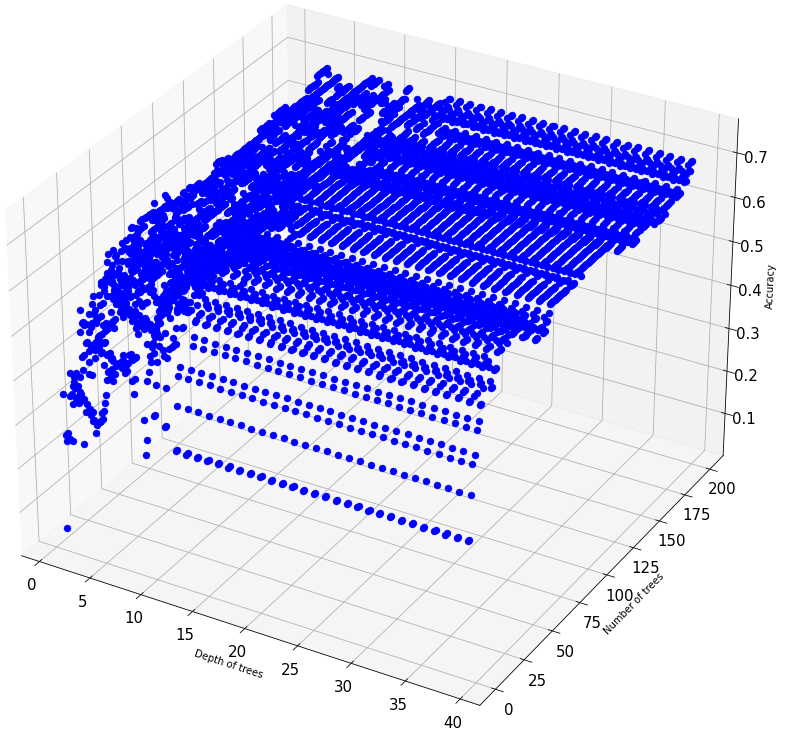

Supplement: S1 Data — (ZIP) [file pone.0276767.s001.zip › Revised/ref4.png]

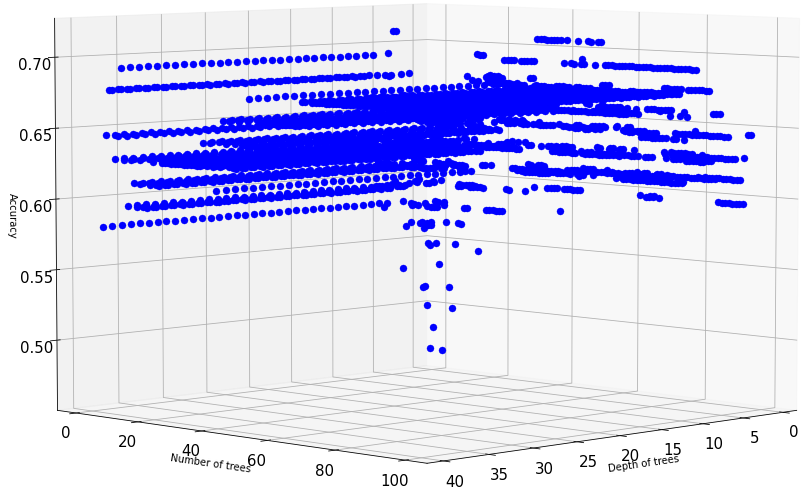

Supplement: S1 Data — (ZIP) [file pone.0276767.s001.zip › Revised/ref6.png]

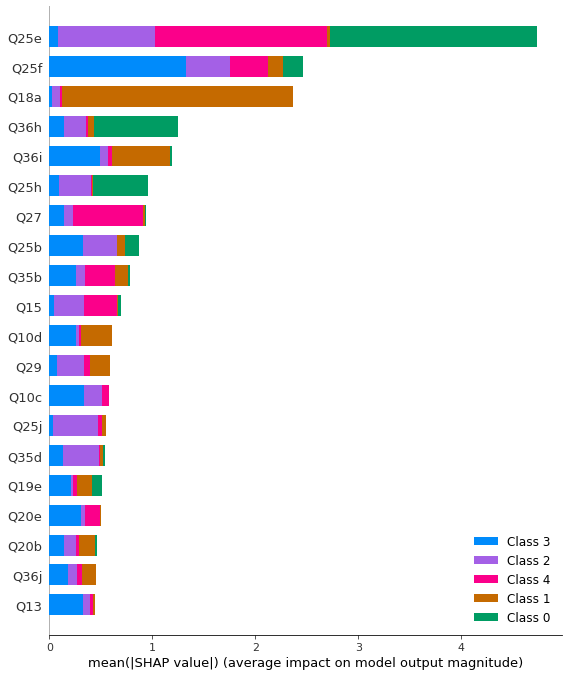

Supplement: S1 Data — (ZIP) [file pone.0276767.s001.zip › Revised/ref7.png]

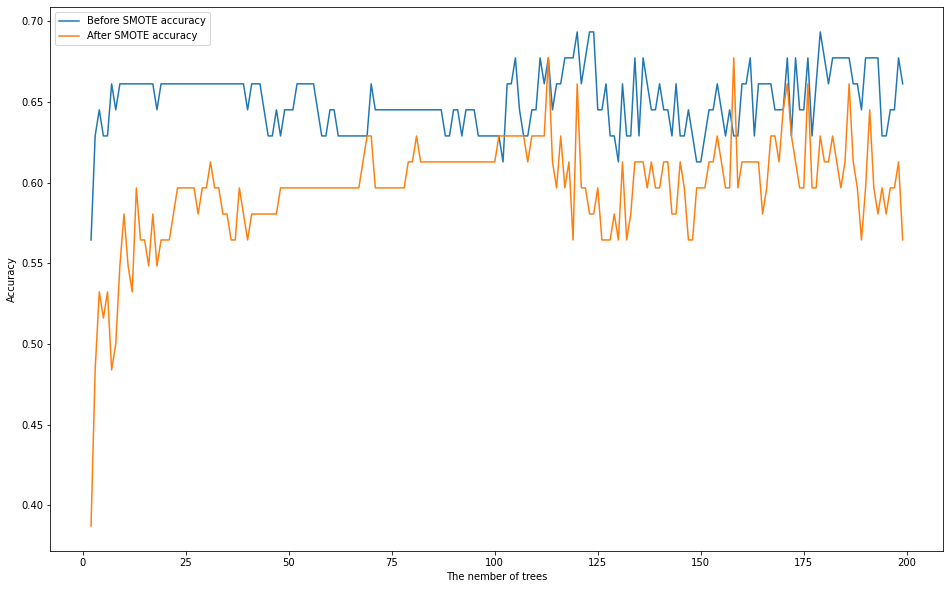

Supplement: S1 Data — (ZIP) [file pone.0276767.s001.zip › Revised/ref8.png]

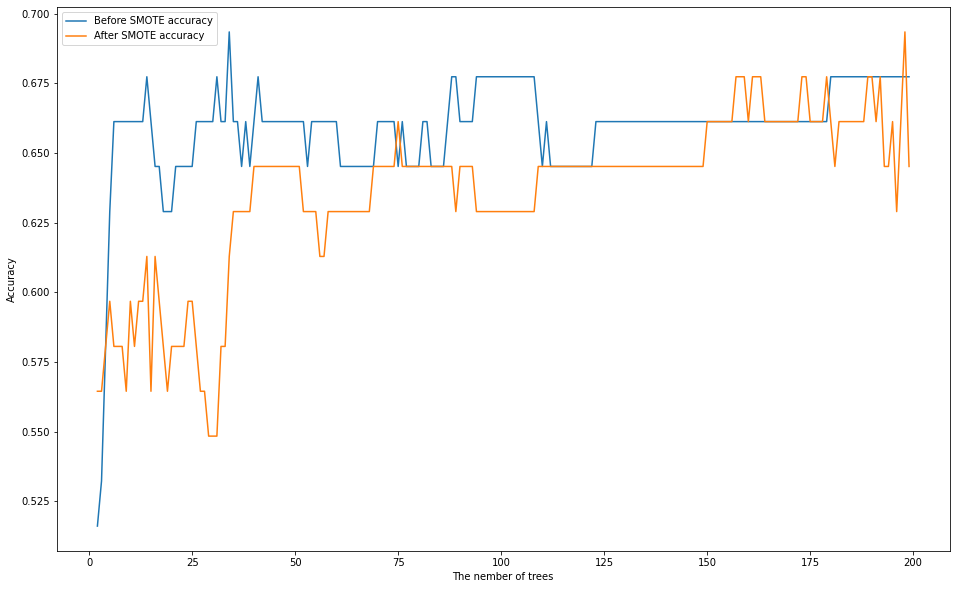

Supplement: S1 Data — (ZIP) [file pone.0276767.s001.zip › Revised/ref9.png]
